# Suppression of overactivated immunity in the early stage is the key to improve the prognosis in severe burns

**DOI:** 10.3389/fimmu.2024.1455899

**Published:** 2024-09-06

**Authors:** Yang Xiang, Bo-han Pan, Jin Zhang, Ji-qiu Chen, He Fang, Qun Wang, Lin-hui Li, Tian-sheng Chen, Jia-xin Chen, Chan Li, Xing-feng Zheng, Shi-hui Zhu

**Affiliations:** ^1^ Department of Burns, Changhai Hospital, Shanghai, China; ^2^ Department of Intensive Care Unit, Shanghai Fourth People’s Hospital Affiliated to Tongji University, Shanghai, China; ^3^ Department of Burns and Plastic Surgery, Shanghai Children’s Medical Center, Shanghai, China

**Keywords:** severe burn, over-activation of immunity, prognosis, S100A8, methotrexate

## Abstract

**Background:**

Severe burns can lead to systemic inflammatory response syndrome (SIRS) and multiple organ dysfunction syndrome (MODS) due to inflammation-immunity dysregulation. This study aimed to identify key immune-related molecules and potential drugs for immune regulation in severe burn treatment.

**Method:**

Microarray datasets GSE77791 and GSE37069 were analyzed to identify immune-related differentially expressed genes (DEGs), enriched pathways and prognosis-related genes. The DGIdb database was used to identify potentially clinically relevant small molecular drugs for hub DEGs. Hub DEGs were validated by total RNA from clinical blood samples through qPCR. The efficacy of drug candidates was tested in a severe burn mouse model. Pathologic staining was used to observe organ damage. Enzyme Linked Immunosorbent Assay (ELISA) was used to detect the serum IL-1b, IL-6, TNF-a and MCP-1 contents. Activation of the NF-κB inflammatory pathway was detected by western blotting. Transcriptome sequencing was used to observe inflammatory-immune responses in the lung.

**Results:**

A total of 113 immune-related DEGs were identified, and the presence of immune overactivation was confirmed in severe burns. S100A8 was not only significantly upregulated and identified to be prognosis-related among the hub DEGs but also exhibited an increasing trend in clinical blood samples. Methotrexate, which targets S100A8, as predicted by the DGIdb, significantly reduces transcription level of S100A8 and inflammatory cytokine content in blood, organ damage (lungs, liver, spleen, and kidneys) and mortality in severely burned mice when combined with fluid resuscitation. The inflammatory-immune response was suppressed in the lungs.

**Conclusion:**

S100A8 with high transcription level in blood is a potential biomarker for poor severe burn prognosis. It suggested that methotrexate has a potential application in severe burn immunotherapy. Besides, it should be emphasized that fluid resuscitation is necessary for the function of methotrexate.

## 1 Introduction

The dangers associated with burns are often underestimated. Approximately 180,000 individuals are estimated to succumb annually to burns and their subsequent complications (1). Immune dysregulation and an inflammatory response are commonly observed in severe burn patients (2, 3).

Existing research indicates that the immune status following severe burn injury can be broadly categorized into three phases: an early phase of immune over-activity, which progresses to immune dysregulation, and ultimately culminates in a late phase of immune suppression (4, 5). In brief, the persistent high-level inflammation and immune hyperactivation in the early stages of severe burns lead to immune exhaustion in both peripheral blood and bone marrow, ultimately inducing immunosuppression (1, 6–12).

However, the main treatment strategies for severe burns such as fluid resuscitation, infection control, wound management and nutrition support, primarily depend on residual physiological recovery potential, which are not proactive interventions for immune dysregulation (1, 13, 14). While immune dysregulation has garnered attention in the clinical management of severe burns, it is often considered primarily as a background factor contributing to the exacerbation of inflammatory responses and the development of sepsis (1).In anti-inflammatory treatment strategies, restoring tissue perfusion and inhibiting the coagulation cascade are the primary therapeutic approaches. As a result, early administration of crystalloid and colloid fluids, along with antithrombin, has become a cornerstone of supportive treatment for patients with severe burns and sepsis (15–17). Ulinastatin may be one of the few immunomodulatory agents utilized in the treatment of severe burns, though it is predominantly used as an adjunct therapy in most cases (1, 18).

Besides, existing clinical biomarkers such as procalcitonin, C-reactive protein, and lactate levels in arterial blood cannot directly reflect immune dysregulation and lack a link to the triggering of SIRS (systemic inflammatory response syndrome) and MODS (multiple organ dysfunction syndrome), though they could reflect the inflammation level and prognosis to some extent (17, 18). Current research primarily focuses on immune abnormalities and key cell types, as well as potential regulatory targets, within the context of infection-induced sepsis (1). However, there remains a need for more targeted studies addressing immune dysregulation, SIRS, and MODS resulting from extensive tissue necrosis in severe burn injuries. Some studies have employed bioinformatics analysis to identify early warning or prognostic biomarkers for severe burns. Notably, transcriptomic data from peripheral blood immune cells have suggested that NFATC2 (Nuclear Factor Of Activated T Cells 2), RORA (RAR Related Orphan Receptor A), and CAMK4 (Calcium Dependent Protein Kinase IV) may serve as prognostic biomarkers for post-burn immune suppression (19). Additionally, analysis of skin samples has indicated that the expression of immune-related genes, and the skin regeneration-related gene could represent critical therapeutic targets for treating burn injuries (20). In related studies validated through *in vitro* experiments, TNF-α (Tumor Necrosis Factor α), the NLRP3 (NLR Family Pyrin Domain Containing 3) inflammasome, D3, neutrophil extracellular traps and PPAR-γ (Peroxisome Proliferator Activated Receptor Gamma) have emerged as significant intervention targets and strategies (21–25).

In contrast to previous studies that have identified infection or sepsis as causes of SIRS, MODS, or immune suppression, this research emphasizes that the early, intense, and prolonged immune over-activity following severe burn injuries is the fundamental cause underlying the development of SIRS, MODS and late-stage immune suppression. In our study, up-regulated S100A8 was identified as a key gene associated with immune dysregulation and prognosis in severe burns through bioinformatics analysis and clinical sample validation. The small molecular drug methotrexate, predicted by S100A8, combined with fluid resuscitation significantly reduced inflammatory cytokine levels, organ damage, and mortality in severely burned mice. Therefore, targeting and suppressing excessive immune over-activity in the early stages of severe burns can reduce the severity of inflammation and inhibit or interrupt the progression of SIRS/MODS. Furthermore, this approach helps to maintain and protect the immune system’s potential, which might have positive implications for later defenses against microbial pathogen invasion.

Overall, for the first time, we highlighted the potential value of immunotherapy as one of the two most necessary major actions in the early stages of severe burns, the other being fluid resuscitation. The study proposes that elevated transcription levels of S100A8 might serve as an early warning indicator for poor prognosis in severe burns. Compared to other small molecular compounds such as Paquinimod, the commonly used drug methotrexate for immune-related diseases has a stronger foundation in evidence-based medicine, making it more secure and valuable for clinical applications. Based on these findings, the study also suggests that methotrexate could be a potential primary agent for immunotherapy in severe burns.

## 2 Method

### 2.1 Data sources

The GSE77791 and GSE37069 datasets were obtained from the GEO database (https://www.ncbi.nlm.nih.gov/geo/). In this study, the GSE77791 dataset was based on the GPL570 [HG-U133_Plus_2] Affymetrix Human Genome U133 Plus 2.0 Array and comprised 30 burn samples collected before treatment and 13 control samples. The GSE37069 dataset was based on chip data from the GPL570 platform and comprised 553 burn samples and 37 control samples. GSE77791 and GSE37069 were used as the training set and external validation set, respectively. A total of 2533 immune-related genes were obtained by combining ImmPort and InnateDB (28, 29).

### 2.2 Identification of DEGs

The R package “limma” was used to identify the DEGs between the burn and control groups in GSE77791 under the criteria of adjusted p value <0.05 and |log2-fold change (FC)|>1 (30). The results were visualized by heatmaps and volcano plots using the “ggplot2” and “pheatmap” R packages, respectively.

### 2.3 WGCNA

WGCNA was performed on the GSE77791 expression data using the R package “WGCNA” (31). First, all samples were checked for outliers to construct a sample clustering tree. The outliers in the sample were then removed based on cutHeight. Then, we chose the best soft-thresholding power β according to standard scale-free networks. Next, co-expression modules were identified by the dynamic pruning method. By WGCNA, the modules most relevant to burns were identified.

### 2.4 Functional enrichment analysis

The intersection of DEGs, key module genes and immune-related genes was determined using the “VennDiagram” R package, and the DEGs were defined as immune-related DEGs and were used for subsequent analysis. GO and KEGG pathway enrichment analyses using the clusterProfiler package were used to explore the functions of immune-related DEGs (32). GO is a powerful tool for analyzing the cellular component (CC), biological function (BP) and molecular function (MF) of immune-related DEGs. The “OmicCircos” R package was utilized to visualize the expression patterns and chromosomal locations of the immune-related DEGs (33).

### 2.5 PPI network construction

The PPI interaction network between the immune-related DEGs was constructed with the STRING database (http://string-db.org) (version 11.5) (34). The hub genes in the PPI networks were identified using the CytoHubba plug-in in Cytoscape (http://www.cytoscape.org) (version 3.9.0). In the Cytohubba plug-in, a total of three algorithms, namely, gene connection degree, maximum neighborhood component (MNC), and maximal clique centrality (MCC), were applied, and the top ten genes were screened. The hub genes were identified by overlapping the top 10 genes of three CytoHubba algorithms.

### 2.6 ROC curve analysis and expression analysis

In the GSE77791 dataset, 30 burn and 13 control samples were utilized to plot ROC curves, from which we obtained their area under the ROC curve (AUC) through the “pROC” package. Good discrimination was defined as an area under the ROC curve (AUC) greater than 0.7. Boxplots of candidate hub gene expression were drawn using “ggplot2” in the R package. The GSE37069 dataset was used to validate the hub genes. When the AUC was > 0.7, the hub was considered to be a key immune-related gene.

### 2.7 Small-molecule drug prediction and GSEA

The key immune-related genes were selected as promising targets for searching for drugs through the Drug Gene Interaction Database (DGIdb) (35). The results were visualized in Cytoscape. The potential functions of the key immune-related genes were analyzed by GSEA. The “clusterprofile” package was utilized to perform GSEA of the key immune-related genes. “C2.cp.kegg.v7.0.symbols.gmt” served as the reference gene set for GSEA.

### 2.8 Establishment of a LASSO logistic regression model

The LASSO model was established using the “glmnet” R package based on the gene expression profiles of key immune-related genes (36). Then, we selected related genes to construct a logistic regression model and constructed a nomogram using the GSE77791 training set. Calibration curves and receiver operating characteristic (ROC) curves were generated to estimate the accuracy and discrimination of the nomogram. Finally, we validated the model using the external validation set GSE37069.

### 2.9 Quantitative real-time PCR

Blood from seven severe burn patients with a TBSA (total body surface area) >30% who entered the care unit from July to September and seven healthy volunteers were sampled in appropriate EDTA-K2 and SST tubes (SKU/REF 368589, BD, US). Red blood cell lysis buffer (R1010, Solarbio, China) was used to isolate leukocytes from whole blood, and total RNA was extracted with a Fast-Pure Cell/Tissue Total RNA Isolation Kit (RC112-01, Vazyme, China). RNA and PrimeScript™ RT Master Mix (Perfect Real Time) (RR036A, Takara, Japan) were used for cDNA synthesis. cDNA and TB Green^®^ Premix Ex Taq™ II (Tli RNaseH Plus) (RR820A, Takara, Japan) were used for qPCR with the primers listed in **Supplementary Table S1**. The 2^−ΔΔCT^ method was used for data quantification.

### 2.10 Different interventions for severe burn mouse model with 72-hour survival curves

C57/bl mice (n=85, eight-week, male; Gempharmatech, China) whose backs were depilated on the previous day were anesthetized with sodium pentobarbital intraperitoneally and fixed. Subsequently, the backs of the mice were immersed in boiling water at 95°C for 4 seconds to construct a severe burn mouse model. The mice were divided into the no fluid resuscitation group (NFR), no fluid resuscitation but methotrexate therapy group (NFRM), fluid resuscitation group (FR), fluid resuscitation combined with methotrexate therapy group (FRM), and control group (NC). All groups consisted of 20 mice, except for the NC group, which consisted of 5 mice. A total of 600 μL of sodium lactate Ringer’s injection was injected intraperitoneally every 12 h after injury as a liquid resuscitation treatment in the FR and FRM groups. MTX (2 μg/kg; HY-14519, MCE, US) was injected intraperitoneally into the NFRM and FRM groups, and the amount of methotrexate solution was considered the total amount of liquid resuscitation. The mice were observed and recorded every 6 hours, and a 72-hour survival curve was constructed.

### 2.11 Enzyme-linked immunosorbent assay

Serum was extracted from the whole blood of mice at 24, 48, and 72 h. The levels of IL-1β, IL-6, TNF-α and MCP-1 were measured using ELISA kits (EK0394, EK0411, EK0527, EK0568, Boster Bio, US). The procedures were in accordance with previous methods (37).

### 2.12 Western blotting analysis

Total protein was extracted with radioimmunoprecipitation buffer (WB3100, NCM Biotech, RIPA) supplemented with protease and phosphatase inhibitors (P6730, P1260, Solarbio, China). Protein concentrations were determined using a Pierce™ BCA Protein Assay Kit (23227, Thermo Fisher, US). An Omni-PAGE™ gel (HEPES 10%, 10 wells) (LK202, Epizyme Biotech, China) was used for protein separation, and proteins were then transferred to PVDF membranes (ISEQ00010, Millipore, US). After blocking with 5% bovine serum albumin, the membranes were washed with TBS plus Tween (TBST) and incubated with primary antibodies against p-IKK (ab194528, Abcam, 1:1000), p-IKBα (ab133462, Abcam, 1:1000), IKBα (ab76429, Abcam, 1:1000), p-p65 (ab76302, Abcam, 1:1000), p65 (ab32536, Abcam, 1:1000), and GAPDH (ab181602, Abcam, 1:1000) overnight, followed by incubation with HRP-conjugated secondary antibodies (LF101, Epizyme Biotech, China, 1:2000) for 1 h. Finally, chemiluminescence was observed on an Amersham Imager 600 multifunctional imaging analysis system (Cytiva, US). All antibodies were diluted in TBST.

### 2.13 Immunohistochemical analysis

Isolated fresh organ samples were sliced into 4-mm thick sections and fixed in 4% paraformaldehyde for 24 h. After dehydration, the samples were embedded in paraffin. Then, the samples were sliced further into 40-μm-thick sections using an SM2000R microtome (Leica, Germany). Sections were deparaffinized in xylene and then hydrated through an alcohol series. HE staining (G1120, Solarbio, China) was performed using hematoxylin to stain the nuclei of the cells to obtain a blue or purple color. After rinsing in clear water, the cytoplasm and other tissue structures were stained with eosin to afford a pink color. The sections were dehydrated with alcohol and cleared using xylene. The sections were sealed for microscopic observation using a sealer.

A semi-quantitative scoring system was used where segments of liver, kidney, spleen and lungs were separately scored for acute inflammation. For renal sections, the degree of tubular injury was scored using the Paller scoring at the severity of the lesion (38). For liver sections, the severity of histopathological changes in the liver was assessed using the Ishak scoring system (39). For lung sections, the Smith Lung Injury Scoring System was used to assess the severity of acute lung injury (40). Enlargement of the B- and T-lymphocyte zones of the spleen (0, none; 1, slight; 2, moderate; 3, marked) was scored. A single value was used for each organ section after separate scoring (minimum value of 0 and maximum value of 6) (41). The score for each tissue sample represents the average of five different high magnification microscopic views.

### 2.14 Transcriptome sequencing analyses

The lungs, one of the earliest and most frequently affected organs in SIRS/MODS, sensitively reflects the state of inflammatory injury (42, 43). Therefore, the lung was selected as a target organ for the assessment of inflammatory injury and immune system activation based on transcriptome sequencing. RNA extraction, purification, reverse transcription, library construction and sequencing were performed at Hangzhou Cosos Wisdom Biotechnology Co., Ltd. (Hangzhou, China) according to the manufacturer’s instructions (Illumina, San Diego, CA). The RNA-seq transcriptome library was prepared following Illumina^®^ Stranded mRNA Prep, Ligation from Illumina (San Diego, CA) using 1 μg of total RNA. Briefly, messenger RNA was first isolated by oligo(dT) beads according to the poly(A) selection method and then fragmented by fragmentation buffer. Second, double-stranded cDNA was synthesized using a SuperScript double-stranded cDNA synthesis kit (Invitrogen, CA) with random hexamer primers (Illumina). Then, the synthesized cDNA was subjected to end repair, phosphorylation and ‘A’ base addition according to Illumina’s library construction protocol. Libraries were size selected for cDNA target fragments of 300 bp on 2% low-range Ultra agarose followed by PCR amplification using Phusion DNA polymerase (NEB) for 15 PCR cycles. After quantification with a Qubit 4.0, the paired-end RNA-seq library was sequenced with a NovaSeq 6000 sequencer (2 × 150 bp read length). After quality control and read mapping, differential expression and functional enrichment analysis were performed.

### 2.15 Statistical analysis

Protein immunoblot images were measured in grey scale using the Analyze-Gels plug-in in image J software. Data were recorded using Microsoft Excel software. The data are presented as the means ± SD. Independent two-sample t tests, one-way analysis of variance and Tukey’s *post hoc* tests (GraphPad Prism; version 9.5; GraphPad Software, Inc.) were used for comparisons between ≥3 groups. P < 0.05 was considered to indicate a statistically significant difference.

## 3 Results

### 3.1 Identification of module genes of burn, DEGs and immune-related DEGs

**Figure 1:**
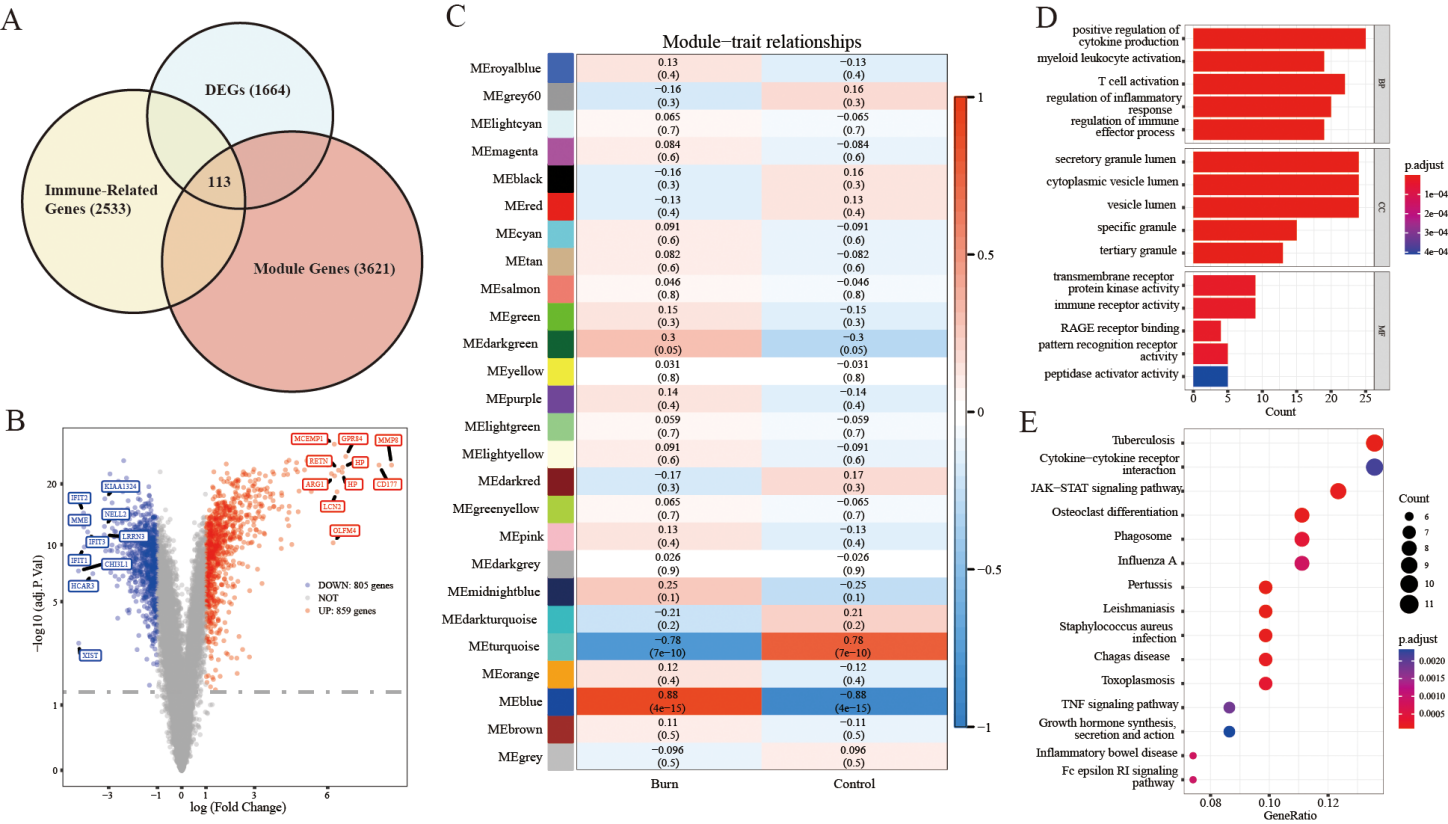
Analyses to obtain immune-related DEGs and functional enrichment. **(A)** Filtering of immune-related DEGs by Venn diagrams with the intersection of total DEGs, module genes and immune-related genes. **(B)** Volcano plot for DEGs between severe burn patients and controls. **(C)** Identification of the module most strongly correlated with burn. **(D, E)** KEGG/GO analysis of immune-related DEGs.

The sample clustering tree indicated that there were no abnormal samples (**Supplementary Figures S1A, B**). By setting the soft-thresholding power to 3 (scale-free R^2^ = 0.85), we eventually identified 25 modules (**Supplementary Figure S1C**). From the heatmap of module-trait correlations, we determined that the blue module was the most highly correlated with burn (correlation coefficient=0.88, p=4e-15; **Figure 1C**). The blue module contained a total of 3621 genes.

A total of 1664 DEGs were screened, which consisted of 859 upregulated genes and 805 downregulated genes (burn vs. control) (**Figure 1B**). A heatmap of the part of the DEGs is shown in **Supplementary Figure S2A**.

We identified 113 immune-related DEGs through the intersection of DEGs, genes in key modules of WGCNA and immune-related genes (**Figure 1A**).

### 3.2 Functional Enrichment of immune-related DEGs

As shown in **Figure 1D**, the GO analysis indicated that immune-related DEGs were mainly enriched in pathways related to the positive regulation of cytokine production, secretory granule lumen, transmembrane receptor protein kinase activity, and immune receptor activity. KEGG analysis was performed to explore the pathways associated with these 113 immune-related DEGs. As shown in **Figure 1E**, KEGG enrichment analysis revealed that these immune-related DEGs were significantly involved in tuberculosis, cytokine−cytokine receptor interactions, and the JAK−STAT signaling pathway. We selected the 113 immune-related DEGs to visualize their expression patterns and chromosomal locations (**Supplementary Figure S2B**). These 113 immune-related DEGs were distributed on chromosomes 1, 2, 3, 4, 5, 6, 7, 8, 9, 10, 11, 12, 13, 14, 15, 17, 19, 20, 21, 22, and X.

### 3.3 Protein−protein interaction network and hub gene identification

**Figure 2:**
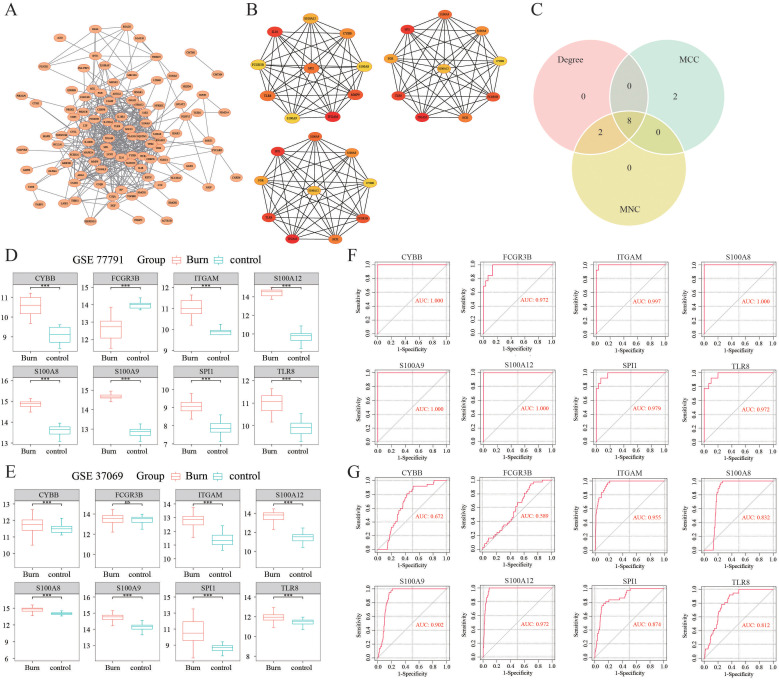
Identification of hub DEGs. **(A)** The PPI network of immune-related DEGs was established using the STRING database. **(B)** The top 10 hub genes selected based on the 3 most commonly used classification methods in CytoHubba. **(C)** Identification of hub DEGs by overlapping the degree, MCC and MNC results. **(D, E)** The expression levels of hub DEGs in the GSE77791 and GSE37069 datasets. **(F, G)** ROC curves of the diagnostic value of the hub DEGs whose AUC values were > 0.7 both in the GSE77791 and GSE37069 datasets. ***: p< 0.001, ns: is non-significant.

A PPI network of immune-related DEGs was constructed using the STRING database (**Figure 2A**). Hub genes were identified by CytoHubba in the present study. The top 10 hub genes, which were selected based on the 3 most commonly used classification methods in cytoHubba, are displayed in **Figure 2B**. By overlapping the top 10 genes identified by the three methods, 8 hub genes (SPI1, TLR8, S100A9, S100A12, S100A8, ITGAM, FCGR3B, and CYBB) were identified, as shown in **Figure 2C**.

### 3.4 Expression analysis and ROC curve analysis of the hub genes

We observed that the gene expression levels of the hub genes, except for FCGR3B, were significantly greater in burn samples than in control samples in the GSE77791 dataset (**Figure 2D**). An external dataset (GSE37069) was used for validation of the expression levels of the 8 hub genes. The results showed that, except for FCGR3B, the expression levels of the remaining seven genes were upregulated, which was consistent with the GSE77791 dataset (**Figure 2E**). As shown in **Figure 2F**, in GSE77791, the AUC values of SPI1, TLR8, S100A9, S100A12, S100A8, ITGAM, FCGR3B, and CYBB were 0.979, 0.972, 1.000, 1.000, 1.000, 0.997, 0.972, and 1.000, respectively, demonstrating that these hub genes had good diagnostic value. In GSE37069, the AUC values of SPI1, TLR8, S100A9, S100A12, S100A8, ITGAM, FCGR3B, and CYBB were 0.874, 0.812, 0.902, 0.972, 0.832, 0.955, 0.589, and 0.672, respectively (**Figure 2G**). Therefore, the six genes of SPI1, TLR8, S100A9, S100A12, S100A8, and ITGAM were identified as the key immune-related genes in burn.

### 3.5 GSEA analyses

**Figure 3:**
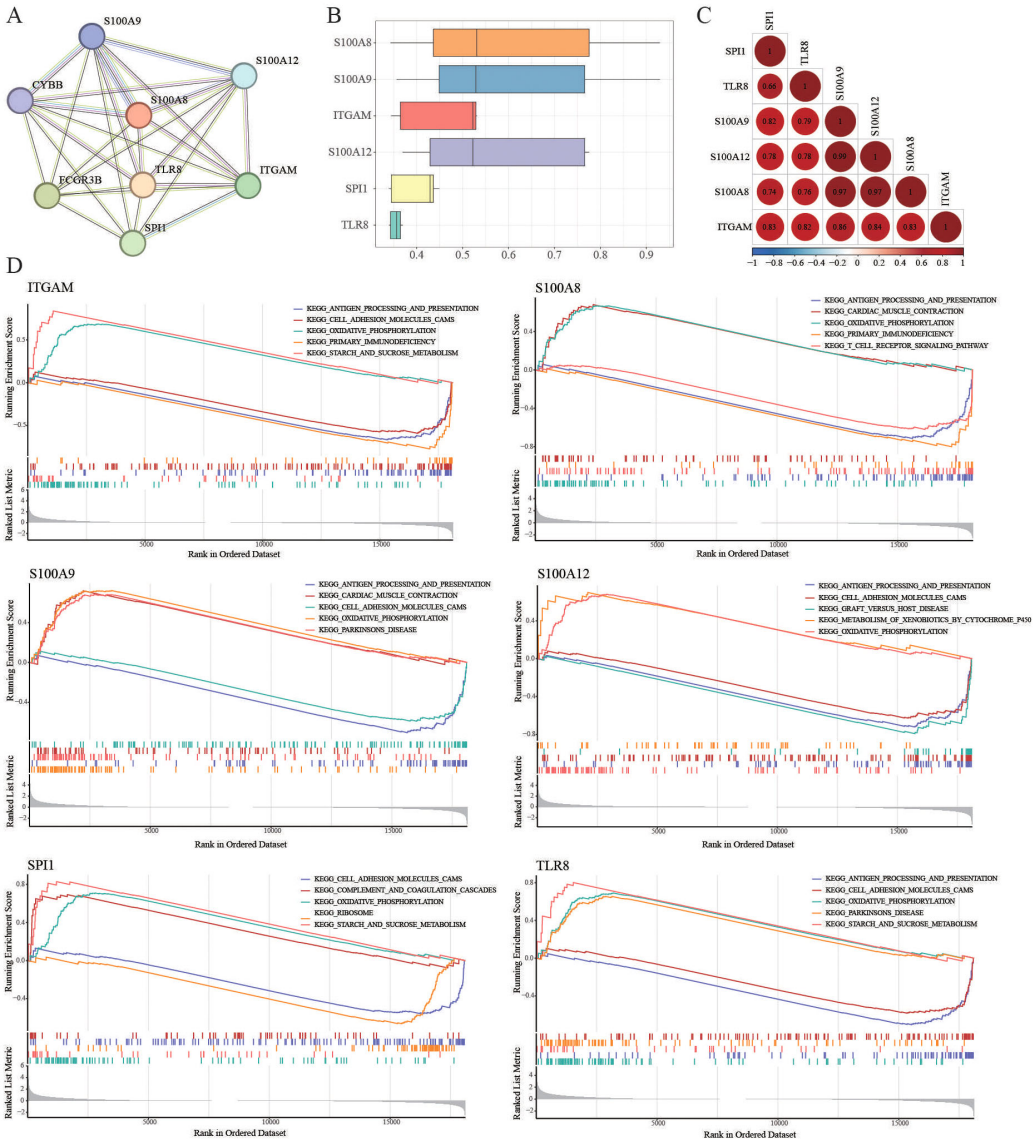
GSEA analyses. **(A)** PPI network of the hub DEGs in the String database. **(B)** GSEA of the functional similarity score. **(C)** The correlation results showed that S100A9 and S100A12 had the strongest positive correlation (r=0.99). **(D)** TLR8, S100A9, S100A12, S100A8, and ITGAM were related to “antigen processing and presentation”. SPI1, TLR8, S100A9, S100A12, S100A8, and ITGAM were associated with “oxidative phosphorylation”.

The PPI network demonstrated strong and complex functional linkages of the hub DEGs in the String Database (**Figure 3A**). First, the functional similarity results showed that S100A8 had the highest functional similarity score (**Figure 3B**). The correlation results showed that S100A9 and S100A12 had the strongest positive correlation (r=0.99, **Figure 3C**). Then, the functions of our key immune-related genes were explored via GSEA (**Figure 3D**). We found that TLR8, S100A9, S100A12, S100A8, and ITGAM were related to “antigen processing and presentation”. SPI1, TLR8, S100A9, S100A12, S100A8, and ITGAM are associated with “oxidative phosphorylation”.

### 3.6 Construction of a survival-related risk regression model

**Figure 4:**
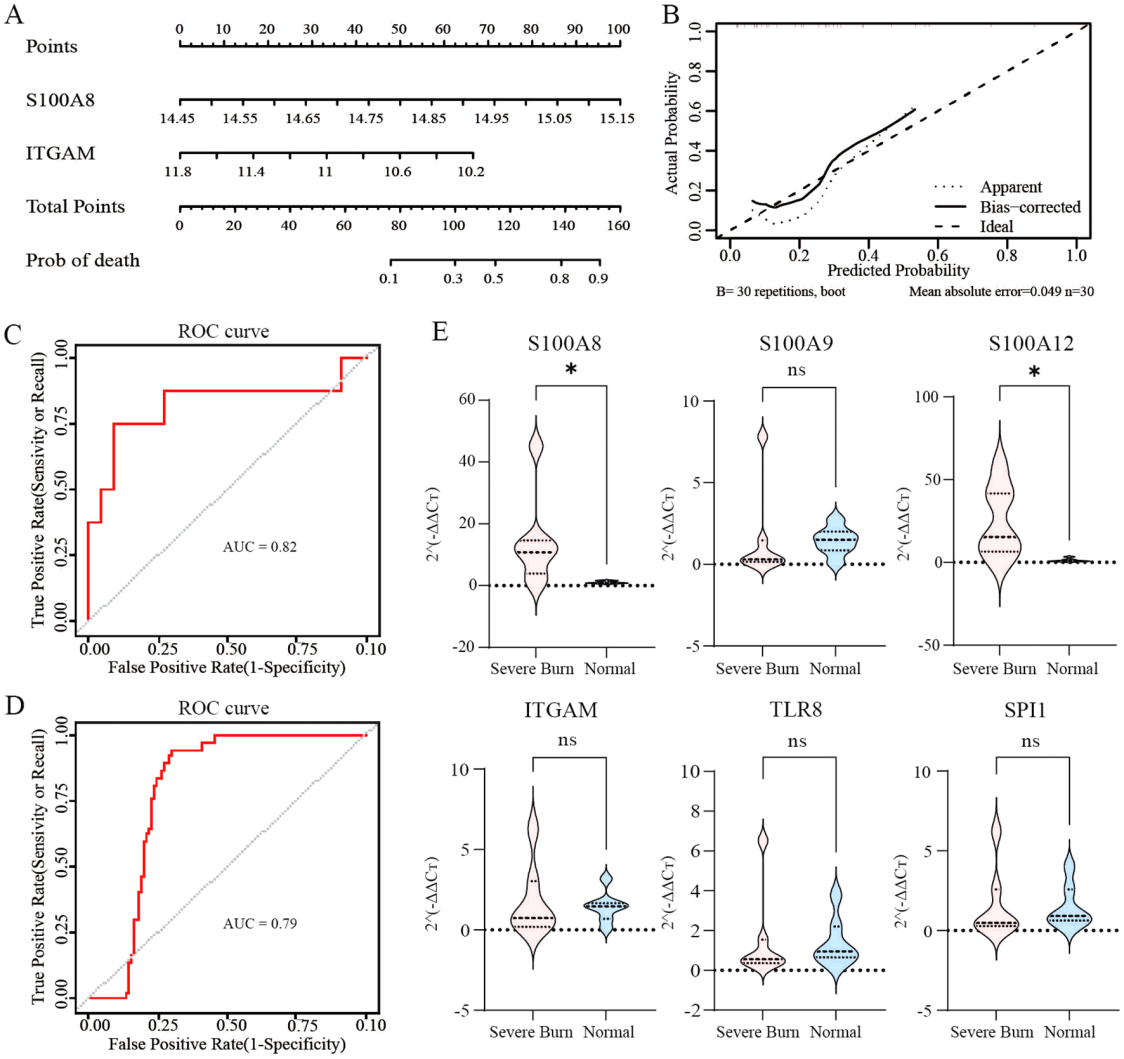
Identification of prognostic molecular and clinical sample validation **(A, B)** Construction of logistic regression models and calibration curves using burn samples (survivors and nonsurvivors) based on S100A8 and ITGAM and visualization via column line plots. **(C, D)** ROC curves with an AUC>0.7 indicated excellent prognostic ability. **(E)** Validation of immune-related hub genes using clinical samples from patients with severe burns. ns: p>0.05 there was no statistic difference between the two groups, *: p< 0.05, versus the normal group.

Thereafter, the LASSO algorithm was used to further screen gene signatures (S100A8 and ITGAM) of burn tissue from key immune-related genes (**Supplementary Figures S3A, B**). Thus, we constructed a logistic regression model using burn samples (survival and nonsurvival) based on S100A8 and ITGAM and constructed a nomogram (**Figure 4A**). **Figure 4B** shows the calibration curves of the model. The area under the curve (AUC) of the ROC curve of the regression model was 0.82 (**Figure 4C**). We measured the model’s prognostic prediction ability in the external cohort (GSE37069), and the area under the curve (AUC) value indicated excellent prediction ability (AUC: 0.79) (**Figure 4D**).

### 3.7 Validation of immune-related hub genes

As depicted in **Figure 4E**, the expression levels of S100A8 and S100A12 in patients with severe burns were significantly greater than those in the control group (S100A8, p=0.0262; S100A12, p=0.0175). However, the differences in the transcription levels of S100A9, ITGAM, TLR8, and SPI1 did not reach statistical significance between the two groups (S100A9, p=0.2086; ITGAM, p=0.62; TLR8, p=0.4557; SPI1, p=0.3829).

### 3.8 Screening of small molecule drugs

**Figure 5:**
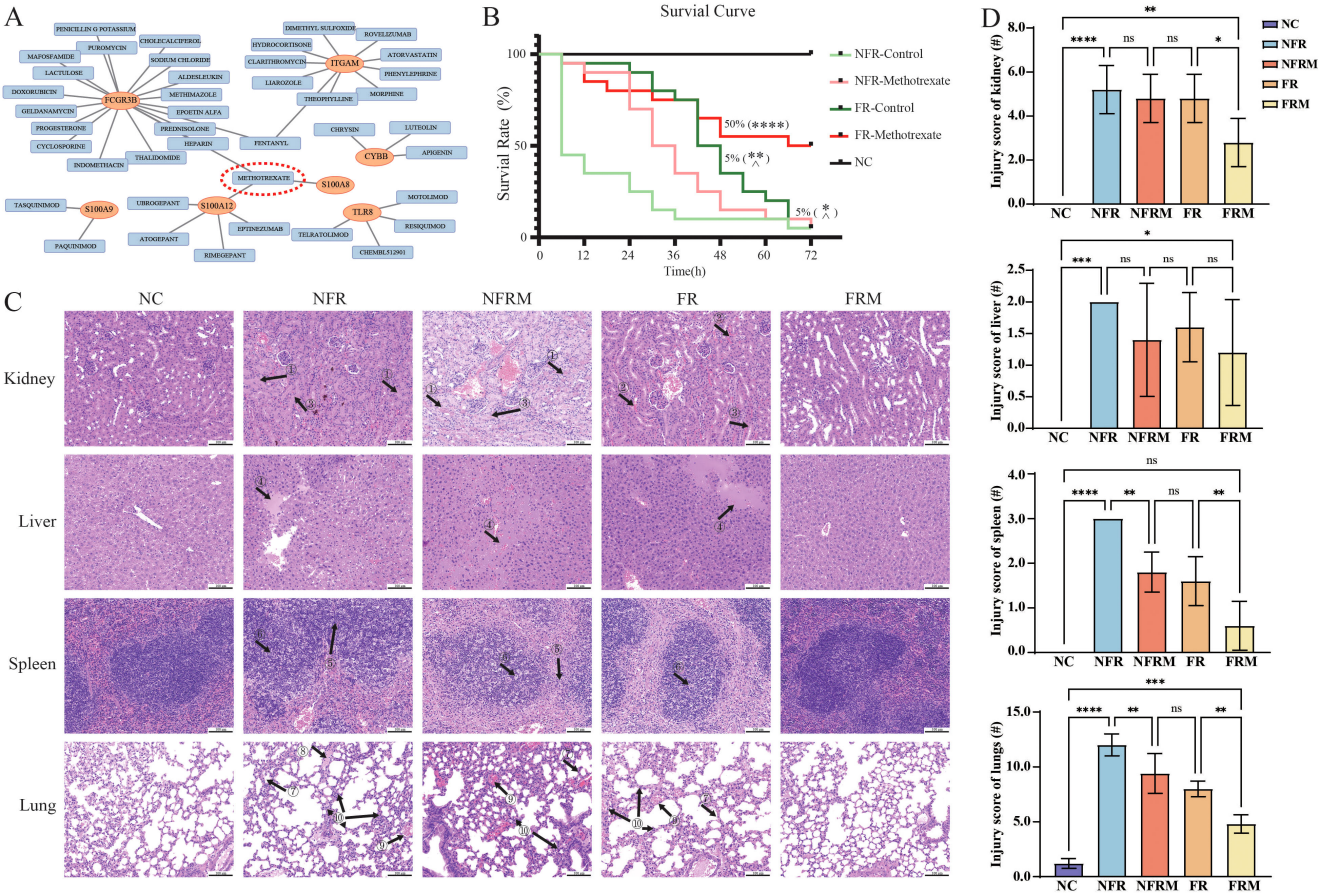
Survival curves and pathological staining in animal experiments. **(A)** Prediction of potential small molecule drugs of immune-related hub genes in the DGIdb database. **(B)** Survival curves of the animals (n=20 in each group; n=5 in sham/NC group) after 72 h. **(C)** HE pathological staining of the lungs, liver, spleen and kidneys of various groups of mice. **(D)** Inflammation injury scoring of kidney, liver, spleen and lungs. ns: p>0.05 there was no statistic difference between the two groups, *: p< 0.05, **: p< 0.01, ***: p< 0.001 and ****: p<0.0001 versus the NFR group. ^: p< 0.05 versus the FRM group. Black arrow with number: ① renal tubular protein tubular pattern; ② renal tubular erythrocyte tubular pattern; ③ renal tubular cell detachment; ④ inflammatory hepatic exudation; ⑤ fusion of splenic lymphocytes in the cortical area; ⑥ lymphoblastoid differentiation; ⑦ alveolar wall thickening; ⑧ hyaline membrane formation; ⑨ alveolar or interstitial hemorrhage; ⑩ lymphocytic or neutrophilic infiltration.

Twenty-one potential drugs for treating burn patients were identified using DGIdb (**Supplementary Table S2**). In this study, 4, 2, 5, 1, and 10 drugs were found to interact with TLR8, S100A9, S100A12, S100A8, and ITGAM, respectively. However, we did not find any small molecule drugs that could target SPI1 in this database. Additionally, drug–gene networks were constructed with Cytoscape (**Figure 5A**).

### 3.9 Survival curves of severe burn mouse model treated with different therapies

The survival curves over a 72-hour period are shown in **Figure 5B**. The mortality rates at 6, 12, 24, 48 and 72 hours for each group were NC (0%), FRM (5%, 15%, 20%, 45%, 50%), FR (5%, 5%, 10%, 65%, 95%), NFRM (5%, 10%, 30%, 85%, 95%), and NFR (55%, 65%, 75%, 90%, and 95%).

According to the log-rank test, the survival curves of FRM (p<0.0001), FR (p=0.0022), and NFRM (p=0.0273) were significantly different from those of NFR, and the survival curves of FR (p=0.0152) and NFRM (p=0.0023) were significantly different from those of FRM. There was no significant difference between the survival curves of FR and NFRM (p=0.1365).

The Gehan-Wilcoxon test showed that when early survival characteristics were taken into account, the survival curves of FRM (p<0.0001), FR (p<0.0001), and NFRM (p=0.0008) were significantly different from those of NFR; there was no significant difference between the survival curves of FR and FRM (p=0.1167); the survival curves of NFRM and FRM were significantly different (p=0.0163); and the survival curves of NFRM and FR were significantly different (p=0.0389).

### 3.10 Pathological findings

HE staining revealed the following pathological changes across the NFR, FR, NFRM groups (**Figure 5C**): (1) Renal tubular protein tubular pattern, erythrocyte tubular pattern, renal tubular cell detachment and collecting duct injury; (2) Inflammatory hepatic exudation and central venous dilatation; (3) Fusion of splenic lymphocytes in the cortical area, lymphoblastoid differentiation; (4) Alveolar wall thickening, hyaline membrane formation, alveolar or interstitial hemorrhage, lymphocytic or neutrophilic infiltration. In terms of injury scores of the four groups (**Figure 5D**), the FRM group had the least organ injury, the NFRM and FR groups had similar levels of injury, and the NFR group had the most severe organ injury.

### 3.11 Transcription level of S100A8 in group FR and FRM

**Figure 6:**
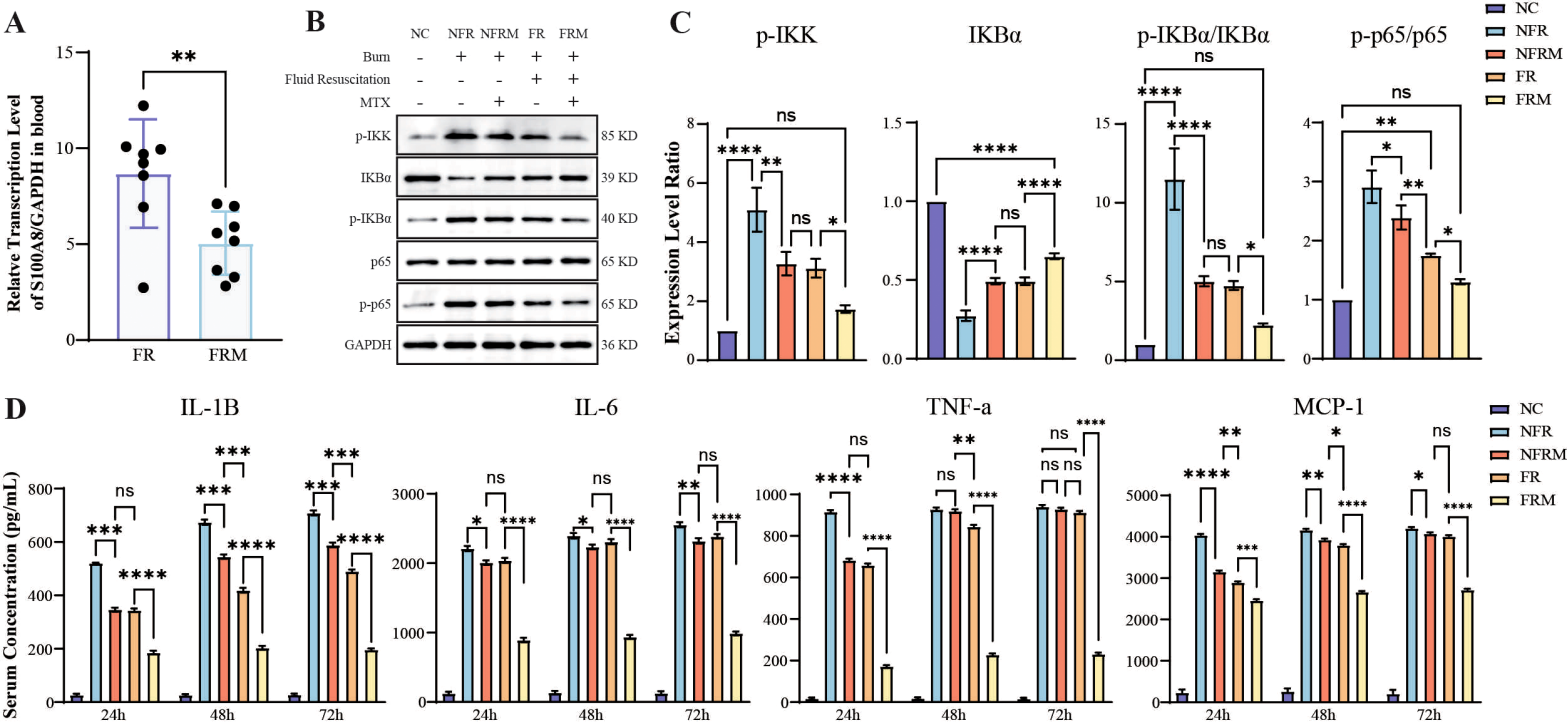
Transcription level of S100A8 in leucocytes, WB and ELISA results for inflammation. **(A)** Transcription level of S100A8 between group FRM and FR. **(B)** Immunoblot images of key proteins of the NF-κB signaling pathway in lung tissue. **(C)** WB analysis of the activation of the NF-κB signaling pathway via the phosphorylation of IKK, IKBα and p65 and the content of IKBα. **(D)** Serum Concentrations of IL-1β, IL-6, TNF-α and MCP-1 tested by ELISA. ns: p>0.05 there was no statistic difference between the two groups, *: p< 0.05, **: p< 0.01, ***: p< 0.001 and ****: p<0.0001 versus the NC group. WB: Western blotting; ELISA: Enzyme-linked immunosorbent assay.

Due to the loss of body fluids as a result of severe burns, only the FR and FRM groups were able to obtain sufficient amounts of RNA for subsequent qPCR to detect the transcript levels of S100A8. As shown in **Figure 6A**, the transcript level of S100A8 in the FRM group (n=8) was significantly lower than that in the FR group (n=8) (p=0.0074). It indicated that methotrexate could effectively reduce the transcript level of S100A8, validating the effectiveness of the predicted drug.

### 3.12 Levels of NF-κB signaling pathway activation

The WB results are shown in **Figure 6B**. The grayscale values of the protein bands were normalized across the groups by using the NC as a baseline “1”. The results indicated that in the FRM group, the levels of p-IKK, the p-IKBα/IKBα ratio, and the p-p65/p65 ratio were significantly lower than those in the NFR, NFRM, and FR groups, while the IKBα content was greater. These findings suggested that the activation level of the NF-κB pathway in the FRM group was lower than that in the NFR, NFRM, and FR groups. In the FR and NFRM groups, the levels of p-IKK, the p-IKBα/IKBα ratio, and the p-p65/p65 ratio were significantly lower than those in the NFR group, while the IKBα content was greater, indicating that the activation level of the NF-κB pathway in the FR and NFRM groups was lower than that in the NFR group. There was no significant difference between the levels of p-IKK and IKBα or the p-IKBα/IKBα ratio between the FR and NFRM groups, except for the p-p65/p65 ratio. This finding suggested that there might be no difference between the NFRM and FR groups in terms of the activation level of the NF-κB pathway.

### 3.13 Cytokine levels

The ELISA results are shown in **Figure 6C**. At all time points, the cytokine levels in the FRM group were significantly lower than those in the FR, NFRM, and NFR groups. At 24 hours, the cytokine levels in the FR and NFRM groups were significantly lower than those in the NFR group. There was no significant difference between the cytokine levels in the FR and NFRM groups, except for those of the IL-1β and TNF-α groups. At 48 hours, the cytokine levels in the FR and NFRM groups, with the exception of TNF-α, were significantly lower than those in the NFR group. There were significant differences in the cytokine levels between the FR and NFRM groups, except for the IL-6 level. At 72 hours, the cytokine levels of the FR and NFRM groups, except for TNF-α, were significantly lower than those of the NFR group, and there was no significant difference between the cytokine levels of the FR and NFRM groups, except for IL-1β.

### 3.14 Transcriptome sequencing findings

**Figure 7:**
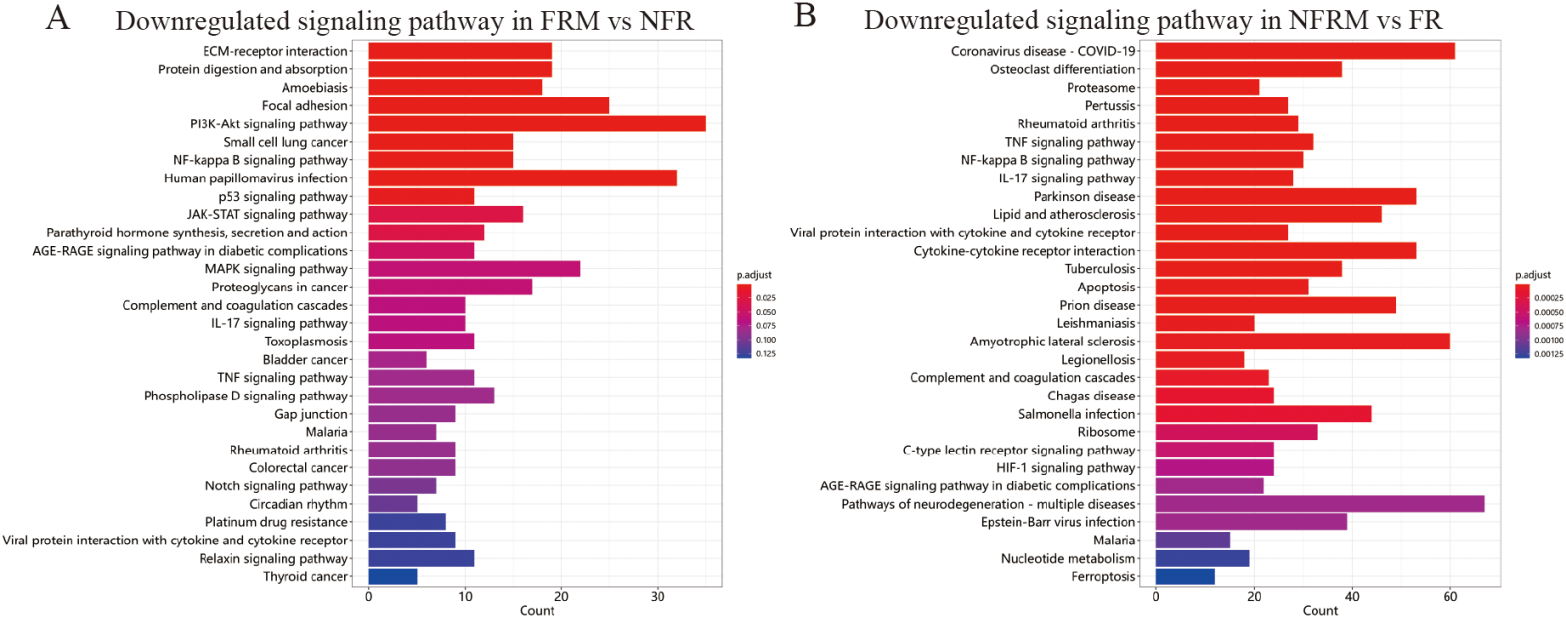
KEGG enrichment of transcriptome sequencing results. **(A)** Downregulated signaling pathways in FRM vs NFR. **(B)** Downregulated signaling pathways in NFRM vs FR.

The GO biological process enrichment analysis plot in **Supplementary Figure S5** shows that compared with FR, FRM downregulated the basic processes of the immune response. Compared to NFRM, FRM upregulated the negative regulation of external stimulus perception and hydrolase activity, as well as the humoral immune response, demonstrating organ-protective effects. Methotrexate significantly downregulated the activation of the JAK-STAT pathway (p=0.029; **Figure 7**).

## 4 Discussion

Overactivated inflammation and immunity devastate the physiological potential and are significantly correlated with poor prognosis (1, 24–26). Through algorithmic screening and clinical sample validation, the transcription level of S100A8 in peripheral blood has been identified as a prognostic marker for patients with severe burns. S100A8 was strongly associated with the cytotoxic effects of neutrophils on acute inflammation in PPI network.

The acute inflammatory response triggered by severe burns is typically accompanied by significant activation and aggregation of neutrophils, which is the initial response of the host’s immune defense mechanism (1, 27). Neutrophil activation in burn injuries is primarily mediated by the release of a series of cytokines and chemokines. These mediators not only promote the migration and aggregation of neutrophils but also enhance their phagocytic activity and reactive oxygen species production, leading to secondary damage at the burn site and distant organs. Recent studies have shown that S100A8 is a crucial calcium-binding protein synthesized and secreted by neutrophils, playing a vital role in calcium homeostasis, apoptosis, and inflammation, and is essential in combating microbial infections and maintaining immune homeostasis. Research indicates that S100A8 mediates inflammation progression via the NLRP3 pathway (28, 29). S100A8 has garnered attention as a potential marker for inflammation and sepsis (30–32). However, the potential of S100A8 to regulate immune dysregulation and improve prognosis in severe burns has not been fully demonstrated. Our study provides a valuable contribution in this area. Huang et al. showed that blocking the binding of S100A8 protein to TLR-4 with Paquinimod reduced mortality in severely burned mice, indirectly supporting the validity of our research (18).

To enhance clinical applicability, our study used the DGIbd database to predict methotrexate as a targeted small-molecule drug for S100A8, based on evidence from evidence-based medicine. Application of methotrexate in severely burned mice significantly reduced the transcription level of S100A8 in peripheral blood, partially proving the predicted targeting efficacy of methotrexate. To date, research on immunosuppressants has focused on autoimmune inflammatory diseases, with limited studies on their application in burns. Achauer and Cetinkale demonstrated that cyclosporine can improve microcirculation, reduce inflammation at the burn site, and increase the survival of skin grafts (33, 34). However, there is no research on cyclosporine’s effects on overall immune dysfunction in burns. Studies have shown that rapamycin can improve acute damage to wounds and organs by inducing autophagy (35–37). Contradictorily, Dunn et al. found that rapamycin increased lung infection risk and reduced survival rates in burned mice (38). Importantly, our bioinformatics analysis revealed that the JAK-STAT signaling pathway is significantly upregulated in severe burn. Methotrexate could sufficiently inhibit pathologic overactivation of the JAK-STAT pathway to control disease and deliver the benefits of specific JAK inhibitors without blocking the physiological activation (39–41). The classical inflammatory JAK-STAT pathway can be activated by pro-inflammatory factors, promoting the expression of inflammatory mediators and further mobilizing immune cells, exacerbating severe reactions. Many studies aim to reduce sepsis inflammation by inhibiting the JAK-STAT signaling pathway (42, 43). However, it must be considered that inhibition of the JAK-STAT pathway can lead to decreased immune cell function, reduced antibody production, cytokine imbalance, and weakened inflammatory response, increasing the risk of inadequate infection control. Clinically, patients treated with JAK inhibitors (e.g., tofacitinib) for autoimmune diseases have shown an increased risk of infection (44, 45). These drugs reduce inflammation and autoimmune responses by inhibiting the JAK-STAT pathway but also lower the body’s immune defense capability, making patients more susceptible to bacterial, viral, and fungal infections. Compared to these immunosuppressants, methotrexate has a relatively mild immunosuppressive effect, helping to retain some immune function for hematopoiesis and the response to infection while controlling the disease (39–41, 46). Although methotrexate has side effects, its severe long-term side effects are relatively fewer and more controllable compared to potent immunosuppressants like cyclophosphamide and corticosteroids. Regular monitoring of liver and kidney function and blood counts can detect and manage potential side effects promptly. Methotrexate can be used in combination with various biologics, enhancing efficacy and reducing the required dosage of biologics, thereby lowering potential side effects.

Additionally, this study explored the application conditions of methotrexate in the treatment of severe burns. The *in-vivo* findings suggest that methotrexate can inhibit immune processes to varying degrees, regardless of fluid resuscitation. Remarkably, methotrexate combined with fluid resuscitation therapy may offer a survival advantage and better inflammation-suppression effect over other treatment modalities, especially methotrexate only. Additionally, the mice in the combined treatment group (FRM) overcame lethargy within 24 hours postinjury and were able to voluntarily consume food and water. And fluid resuscitation alone had a greater effect than methotrexate alone in the first 48 h, although this approach did not significantly alter the mortality rate within 72 hours postburn in mice. Although the expression of inflammatory factors was reduced by methotrexate, they would still accumulate in the circulation gradually without fluid resuscitation to be eliminated. Therefore, the poor prognostic outcome may not be effectively improved. In contrast, a stable circulatory volume combined with methotrexate successfully improved the condition of severely burned mice. Thus, we emphasized the necessity of fluid resuscitation as a cornerstone for application of methotrexate in severe burn.

Overall, these results demonstrate that the combination of methotrexate and fluid resuscitation can significantly inhibit excessive immune activation in the early stages of severe burns, effectively maintaining immune and inflammatory responses to preserve organ function. This significant protective effect on organ function markedly improves the prognosis of severely burned mice.

The multivariate analysis did not include variables that may confound the prediction of severe burns, such as preexisting nutritional and immune status, comorbidities, duration of burn wound excision, or further supportive therapeutic measures. In addition, methotrexate has a wide range of downstream sites, and its interaction with S100A8 needs to be further clarified. Therefore, more multicenter clinical trials and *in vitro* studies are necessary to gain a comprehensive understanding of immune modulation for the treatment of severe burns and to elucidate the underlying cellular and molecular mechanisms involved.

## 5 Conclusion

This study provides insight into the role of immune-related molecules in severe burns and examines the impact of targeted small molecule drugs on the development and severity of SIRS/MODS. The starting point of our study was to screen for S100A8, which is strongly associated with neutrophil activity and prognosis. After validation with clinical samples, S100A8 was predicted to yield the targeted clinical small-molecule drug methotrexate. Combining methotrexate with fluid resuscitation resulted in reduction of severe burn mortality, attenuation of immune and inflammatory responses, and organ damage in severely burned mice.

## Data Availability

The original contributions presented in the study are included in the article/**Supplementary Material**. The mouse tissue transcriptome sequencing data involved in the study have been uploaded to the National Genomics Data Center (https://www.cncb.ac.cn/; BioProject Number: PRJCA029404; GSA Submission Number: CRA018530). The data is expected to be released in September 2024. Further inquiries can be directed to the corresponding authors

## Supplementary material

Supplementary Figure 1 Construction of co-expression networks. **(A)** The sample clustering tree indicated that there were no abnormal samples. **(B, C)** Twenty-five gene modules were identified by setting the soft-thresholding power to 3 (scale-free R^2 =^ 0.85) in WGCNA.

Supplementary Figure 2 Heatmap and distribution on chromosomes of DEGs. **(A)** Part of DEGs were screened in heatmap. **(B)** These 113 immune-related DEGs were distributed on chromosomes 1, 2, 3, 4, 5, 6, 7, 8, 9, 10, 11, 12, 13, 14, 15, 17, 19, 20, 21, 22, and X.

Supplementary Figure 3 Construction of a survival-related risk regression model. **(A, B)** LASSO algorithm was to further screen gene signature (S100A8 and ITGAM) of burn from key immune-related genes.

Supplementary Figure 4 Pathological staining. HE pathological staining images (100X to original magnification) of the lungs, liver, spleen and kidneys of various groups of mice. Black arrow with number: ① renal collecting duct injury; ② inflammatory hepatic exudation; ③ hepatic central venous dilatation; ④ fusion of splenic lymphocytes in the cortical area.

Supplementary Figure 5 Transcriptome sequencing results. **(A)** GO functional enrichment analysis of biological processes in the FRM versus FR groups. The left panel shows genes whose expression increased, and the right panel shows genes whose expression decreased. **(B)** GO functional enrichment analysis of biological process terms associated with FRM versus NFRM. The left figure shows the upregulation process, and the right figure shows the downregulation process.

## References

[1] Jeschke MG van Baar ME Choudhry MA Chung KK Gibran NS Logsetty S. Burn injury. Nat Rev Dis Primers. (2020) 6:11. doi: 10.1038/s41572-020-0145-5 32054846 PMC7224101

[2] Zhao G Yu YM Kaneki M Bonab AA Tompkins RG Fischman AJ. Simvastatin reduces burn injury-induced splenic apoptosis via downregulation of the TNF-alpha/NF-kappaB pathway. Ann Surg. (2015) 261:1006–12. doi: 10.1097/SLA.0000000000000764 PMC4272342 24950285

[3] Johnson BZ McAlister S McGuire HM Palanivelu V Stevenson A Richmond P. Pediatric burn survivors have long-term immune dysfunction with diminished vaccine response. Front Immunol. (2020) 11:1481. doi: 10.3389/fimmu.2020.01481 32793203 PMC7385079

[4] Rogobete AF Sandesc D Papurica M Stoicescu ER Popovici SE Bratu LM. The influence of metabolic imbalances and oxidative stress on the outcome of critically ill polytrauma patients: a review. Burns Trauma. (2017) 5:8. doi: 10.1186/s41038-017-0073-0 28286784 PMC5341432

[5] Rae L Fidler P Gibran N. The physiologic basis of burn shock and the need for aggressive fluid resuscitation. Crit Care Clin. (2016) 32:491–505. doi: 10.1016/j.ccc.2016.06.001 27600122

[6] Sayyadioskoie SR Schwacha MG. Myeloid-derived suppressor cells (MDSCs) and the immunoinflammatory response to injury (Mini review). Shock. (2021) 56:658–66. doi: 10.1097/SHK.0000000000001795 33882515

[7] Cuenca AG Delano MJ Kelly-Scumpia KM Moreno C Scumpia PO Laface DM. A paradoxical role for myeloid-derived suppressor cells in sepsis and trauma. Mol Med. (2011) 17:281–92. doi: 10.2119/molmed.2010.00178 PMC3060988 21085745

[8] Yi M Li T Niu M Mei Q Zhao B Chu Q. Exploiting innate immunity for cancer immunotherapy. Mol Cancer. (2023) 22:187. doi: 10.1186/s12943-023-01885-w 38008741 PMC10680233

[9] Gabrilovich DI Nagaraj S. Myeloid-derived suppressor cells as regulators of the immune system. Nat Rev Immunol. (2009) 9:162–74. doi: 10.1038/nri2506 PMC2828349 19197294

[10] Cheng Z Abrams ST Toh J Wang SS Wang Z Yu Q. The critical roles and mechanisms of immune cell death in sepsis. Front Immunol. (2020) 11:1918. doi: 10.3389/fimmu.2020.01918 32983116 PMC7477075

[11] Sikora JP Karawani J Sobczak J. Neutrophils and the systemic inflammatory response syndrome (SIRS). Int J Mol Sci. (2023) 24:13469. doi: 10.3390/ijms241713469 37686271 PMC10488036

[12] Shafqat A Khan JA Alkachem AY Sabur H Alkattan K Yaqinuddin A. How neutrophils shape the immune response: reassessing their multifaceted role in health and disease. Int J Mol Sci. (2023) 24:17583. doi: 10.3390/ijms242417583 38139412 PMC10744338

[13] Greenhalgh DG. Management of burns. N Engl J Med. (2019) 380:2349–59. doi: 10.1056/NEJMra1807442 31189038

[14] Salyer CE Bomholt C Beckmann N Bergmann CB Plattner CA Caldwell CC. Novel therapeutics for the treatment of burn infection. Surg Infect (Larchmt). (2021) 22:113–20. doi: 10.1089/sur.2020.104 PMC7826423 32429749

[15] Gurney JM Kozar RA Cancio LC. Plasma for burn shock resuscitation: is it time to go back to the future? Transfusion. (2019) 59:1578–86. doi: 10.1111/trf.15243 30980739

[16] Cotton BA Guy JS Morris JA Jr Abumrad NN. The cellular, metabolic, and systemic consequences of aggressive fluid resuscitation strategies. Shock. (2006) 26:115–21. doi: 10.1097/01.shk.0000209564.84822.f2 16878017

[17] Iba T Saitoh D. Efficacy of antithrombin in preclinical and clinical applications for sepsis-associated disseminated intravascular coagulation. J Intensive Care. (2014) 2:66. doi: 10.1186/s40560-014-0051-6 25705422 PMC4336274

[18] Li J Hu C Yang H Yao Y. Effects of ulinastatin on immune function of patients with severe burn injury. Zhonghua Shao Shang Za Zhi. (2016) 32:345–50. doi: 10.3760/cma.j.issn.1009-2587.2016.06.009 27321488

[19] Wang P Zhang Z Yin B Li J Xialin C Lian W. Identifying changes in immune cells and constructing prognostic models using immune-related genes in post-burn immunosuppression. PeerJ. (2022) 10:e12680. doi: 10.7717/peerj.12680 35070500 PMC8761370

[20] Huang J Chen Y Guo Z Yu Y Zhang Y Li P. Prospective study and validation of early warning marker discovery based on integrating multi-omics analysis in severe burn patients with sepsis. Burns Trauma. (2023) 11:tkac050. doi: 10.1093/burnst/tkac050 36659877 PMC9840905

[21] Long H Xu B Luo Y Luo K. Artemisinin protects mice against burn sepsis through inhibiting NLRP3 inflammasome activation. Am J Emerg Med. (2016) 34:772–7. doi: 10.1016/j.ajem.2015.12.075 26830216

[22] Tsay TB Yang MC Chen PH Lai KH Huang HT Hsu CM. Blocking TNF-α enhances Pseudomonas aeruginosa-induced mortality in burn mice through induction of IL-1β. Cytokine. (2013) 63:58–66. doi: 10.1016/j.cyto.2013.04.002 23623770

[23] Chen Y Guo JH Chen YJ Huang Y Zhang C Zhang Q. 1,25-Dihydroxyvitamin D3 reduces early mortality post severe burn injury via alleviating endotoxemia, oxidative stress and inflammation. Burns. (2024) 8:S0305-4179(24)00151-7. doi: 10.1016/j.burns.2024.05.009 38987082

[24] Elrod J Lenz M Kiwit A Armbrust L Schönfeld L Reinshagen K. Murine scald models characterize the role of neutrophils and neutrophil extracellular traps in severe burns. Front Immunol. (2023) 14:1113948. doi: 10.3389/fimmu.2023.1113948 36825027 PMC9941538

[25] Taira BR Singer AJ McClain SA Lin F Rooney J Zimmerman T. Rosiglitazone, a PPAR-gamma ligand, reduces burn progression in rats. J Burn Care Res. (2009) 30:499–504. doi: 10.1097/BCR.0b013e3181a28e37 19349877

[26] Liu D Huang SY Sun JH Zhang HC Cai QL Gao C. Sepsis-induced immunosuppression: mechanisms, diagnosis and current treatment options. Mil Med Res. (2022) 9:56. doi: 10.1186/s40779-022-00422-y 36209190 PMC9547753

[27] Barichello T Generoso JS Singer M Dal-Pizzol F. Biomarkers for sepsis: more than just fever and leukocytosis-a narrative review. Crit Care. (2022) 26:14. doi: 10.1186/s13054-021-03862-5 34991675 PMC8740483

[28] Bhattacharya S Dunn P Thomas CG Smith B Schaefer H Chen J. ImmPort, toward repurposing of open access immunological assay data for translational and clinical research. Sci Data. (2018) 5:180015. doi: 10.1038/sdata.2018.15 29485622 PMC5827693

[29] Breuer K Foroushani AK Laird MR Chen C Sribnaia A Lo R. InnateDB: systems biology of innate immunity and beyond–recent updates and continuing curation. Nucleic Acids Res. (2013) 41:D1228–33. doi: 10.1093/nar/gks1147 PMC3531080 23180781

[30] Ritchie ME Phipson B Wu D Hu Y Law CW Shi W. limma powers differential expression analyses for RNA-sequencing and microarray studies. Nucleic Acids Res. (2015) 43:e47. doi: 10.1093/nar/gkv007 25605792 PMC4402510

[31] Langfelder P Horvath S. WGCNA: an R package for weighted correlation network analysis. BMC Bioinf. (2008) 9:559. doi: 10.1186/1471-2105-9-559 PMC2631488 19114008

[32] Yu G Wang LG Han Y He QY. clusterProfiler: an R package for comparing biological themes among gene clusters. OMICS. (2012) 16:284–7. doi: 10.1089/omi.2011.0118 PMC3339379 22455463

[33] Hu Y Yan C Hsu CH Chen QR Niu K Komatsoulis GA. OmicCircos: A simple-to-use R package for the circular visualization of multidimensional omics data. Cancer Inform. (2014) 13:13–20. doi: 10.4137/CIN.S13495 PMC3921174 24526832

[34] Szklarczyk D Franceschini A Wyder S Forslund K Heller D Huerta-Cepas J. STRING v10: protein-protein interaction networks, integrated over the tree of life. Nucleic Acids Res. (2015) 43:D447–52. doi: 10.1093/nar/gku1003 PMC4383874 25352553

[35] Cotto KC Wagner AH Feng YY Kiwala S Coffman AC Spies G. DGIdb 3.0: a redesign and expansion of the drug-gene interaction database. Nucleic Acids Res. (2018) 46:D1068–73. doi: 10.1093/nar/gkx1143 PMC5888642 29156001

[36] Engebretsen S Bohlin J. Statistical predictions with glmnet. Clin Epigenet. (2019) 11:123. doi: 10.1186/s13148-019-0730-1 PMC6708235 31443682

[37] Feng Y Mei L Wang M Huang Q Huang R. Anti-inflammatory and pro-apoptotic effects of 18beta-glycyrrhetinic acid in vitro and in vivo models of rheumatoid arthritis. Front Pharmacol. (2021) 12:681525. doi: 10.3389/fphar.2021.681525 34381358 PMC8351798

[38] Chang Y Han Z Zhang Y Zhou Y Feng Z Chen L. G protein-coupled estrogen receptor activation improves contractile and diastolic functions in rat renal interlobular artery to protect against renal ischemia reperfusion injury. BioMed Pharmacother. (2019) 112:108666. doi: 10.1016/j.biopha.2019.108666 30784936

[39] Sanyal AJ Anstee QM Trauner M Lawitz EJ Abdelmalek MF Ding D. Cirrhosis regression is associated with improved clinical outcomes in patients with nonalcoholic steatohepatitis. Hepatology. (2022) 75:1235–46. doi: 10.1002/hep.32204 PMC9303958 34662449

[40] Shimizu J Murao A Mofi C Wang P Aziz M. Extracellular CIRP promotes GPX4-mediated ferroptosis in sepsis. Front Immunol. (2022) 13:903859. doi: 10.3389/fimmu.2022.903859 35844517 PMC9277504

[41] Giamarellos-Bourboulis EJ Tziortzioti V Koutoukas P Baziaka F Raftogiannis M Antonopoulou A. Clarithromycin is an effective immunomodulator in experimental pyelonephritis caused by pan-resistant Klebsiella pneumoniae. J Antimicrob Chemother. (2006) 57:937–44. doi: 10.1093/jac/dkl084 16549515

[42] Roy SK Kendrick D Sadowitz BD Gatto L Snyder K Satalin JM. Jack of all trades: pleiotropy and the application of chemically modified tetracycline-3 in sepsis and the acute respiratory distress syndrome (ARDS). Pharmacol Res. (2011) 64:580–9. doi: 10.1016/j.phrs.2011.06.012 PMC3195907 21767646

[43] Hoyer FF Naxerova K Schloss MJ Hulsmans M Nair AV Dutta P. Tissue-specific macrophage responses to remote injury impact the outcome of subsequent local immune challenge. Immunity. (2019) 51:899–914.e7. doi: 10.1016/j.immuni.2019.10.010 31732166 PMC6892583

[44] Serra Lopez-Matencio JM Vicente-Rabaneda EF Alañón E Aranguren Oyarzabal A Martínez Fleta P Castañeda S. COVID-19 vaccination and immunosuppressive therapy in immune-mediated inflammatory diseases. Vaccines (Basel). (2023) 11(12):1813. doi: 10.3390/vaccines11121813 38140217 PMC10747214

[45] Venetsanopoulou AI Voulgari PV Drosos AA. Advances in non-biological drugs for the treatment of rheumatoid arthritis. Expert Opin Pharmacother. (2023) 25(1):45–53. doi: 10.1080/14656566.2023.2297798 38126739

[46] Beinlich FRM Asiminas A Untiet V Bojarowska Z Plá V Sigurdsson B. Oxygen imaging of hypoxic pockets in the mouse cerebral cortex. Science. (2024) 383:1471–8. doi: 10.1126/science.adn1011 PMC11251491 38547288

