# Supplementary figures and images for: Suppression of overactivated immunity in the early stage is the key to improve the prognosis in severe burns
Source: Front Immunol. 2024 Sep 6;15:1455899. doi: 10.3389/fimmu.2024.1455899 (PMC11412824; doi:10.3389/fimmu.2024.1455899)

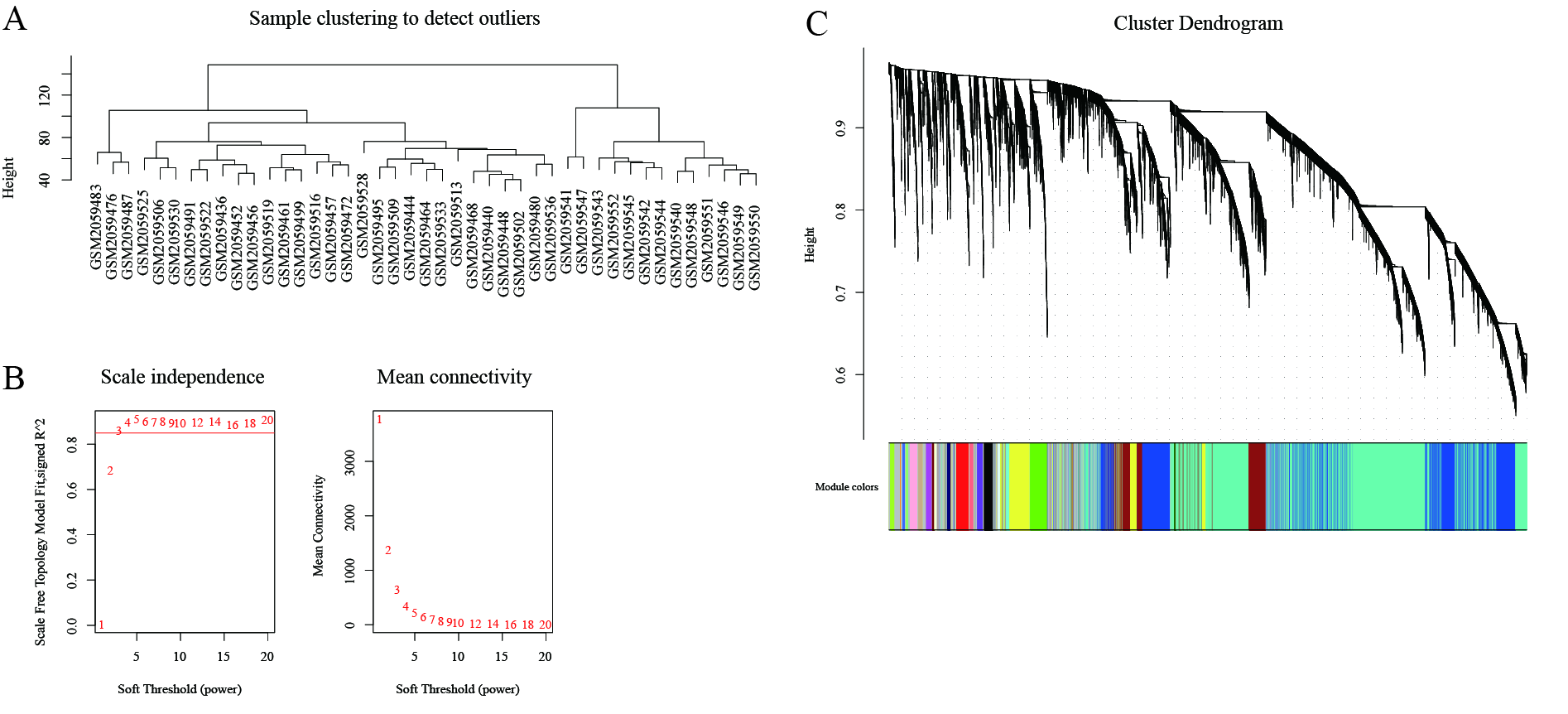

Supplement: Supplementary Figure 1 — Construction of co-expression networks. (A) The sample clustering tree indicated that there were no abnormal samples. (B, C) Twenty-five gene modules were identified by setting the soft-thresholding power to 3 (scale-free R2 = 0.85) in WGCNA. [file Image1.tif]

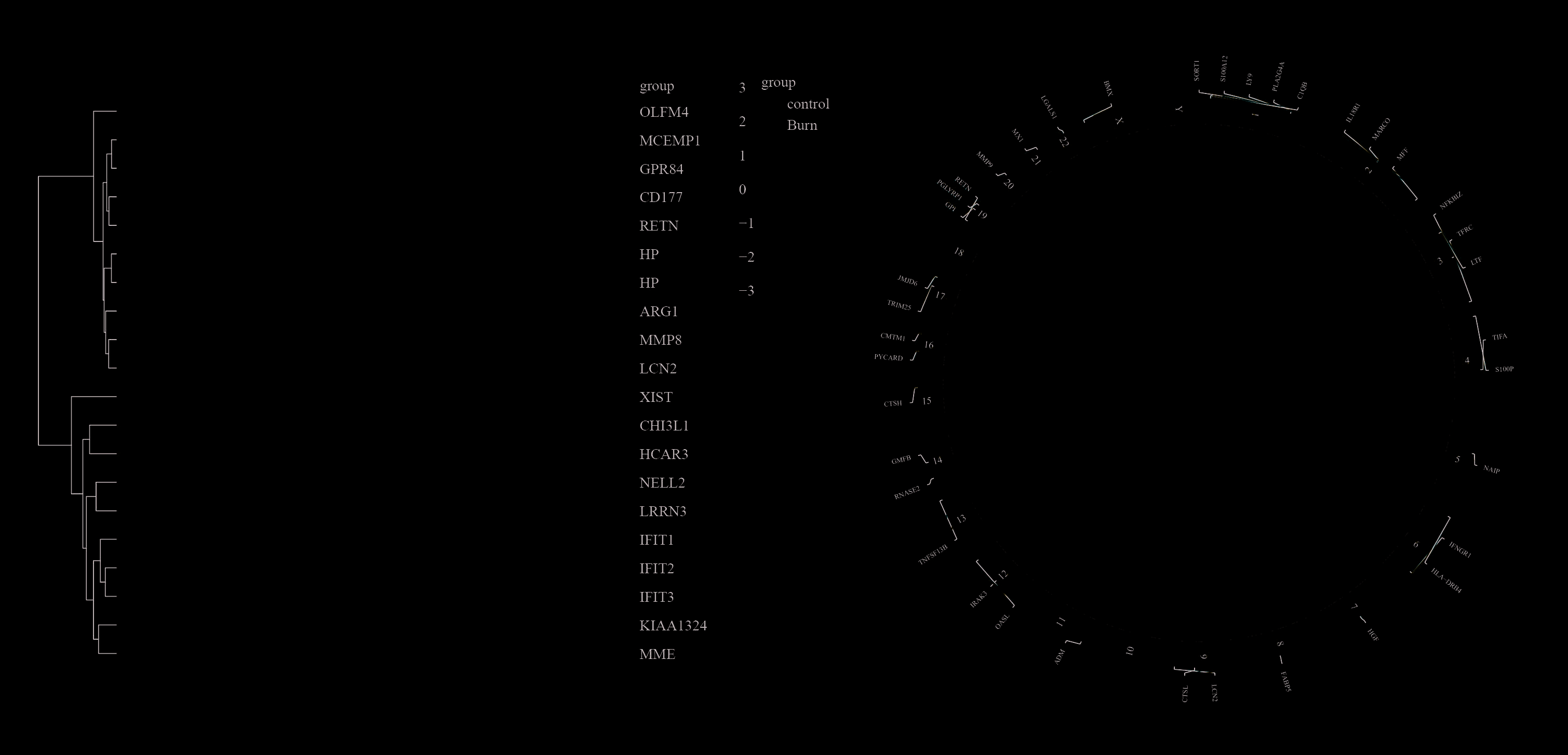

Supplement: Supplementary Figure 2 — Heatmap and distribution on chromosomes of DEGs. (A) Part of DEGs were screened in heatmap. (B) These 113 immune-related DEGs were distributed on chromosomes 1, 2, 3, 4, 5, 6, 7, 8, 9, 10, 11, 12, 13, 14, 15, 17, 19, 20, 21, 22, and X. [file Image2.tif]

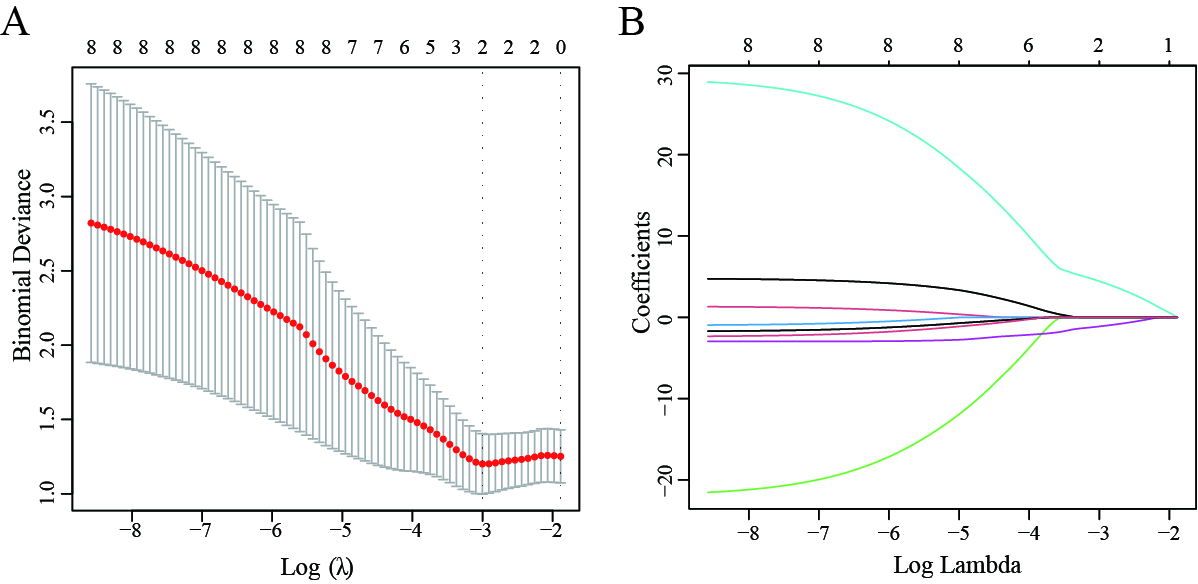

Supplement: Supplementary Figure 3 — Construction of a survival-related risk regression model. (A, B) LASSO algorithm was to further screen gene signature (S100A8 and ITGAM) of burn from key immune-related genes. [file Image3.tif]

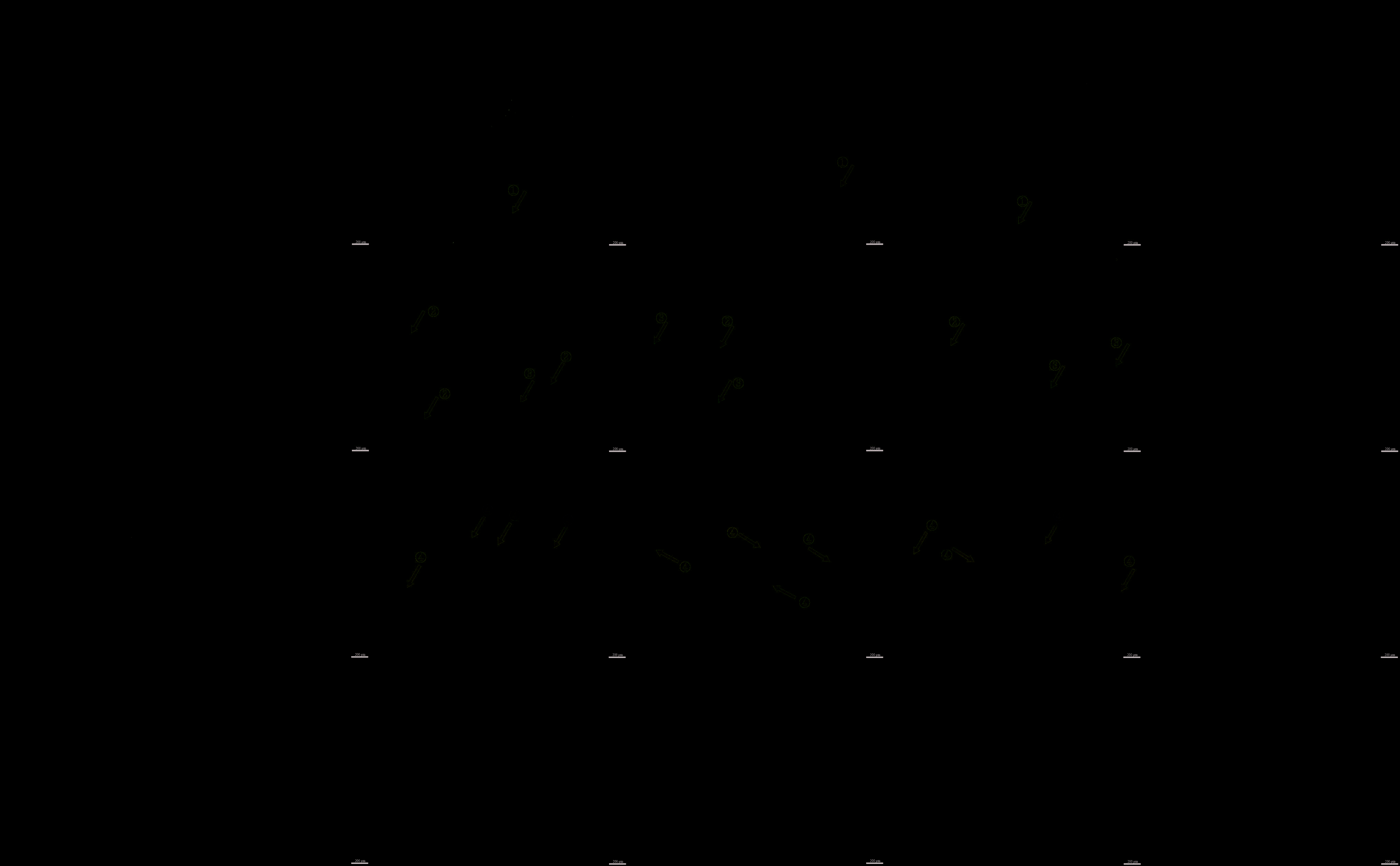

Supplement: Supplementary Figure 4 — Pathological staining. HE pathological staining images (100X to original magnification) of the lungs, liver, spleen and kidneys of various groups of mice. Black arrow with number: ① renal collecting duct injury; ② inflammatory hepatic exudation; ③ hepatic central venous dilatation; ④ fusion of splenic lymphocytes in the cortical area. [file Image4.tif]

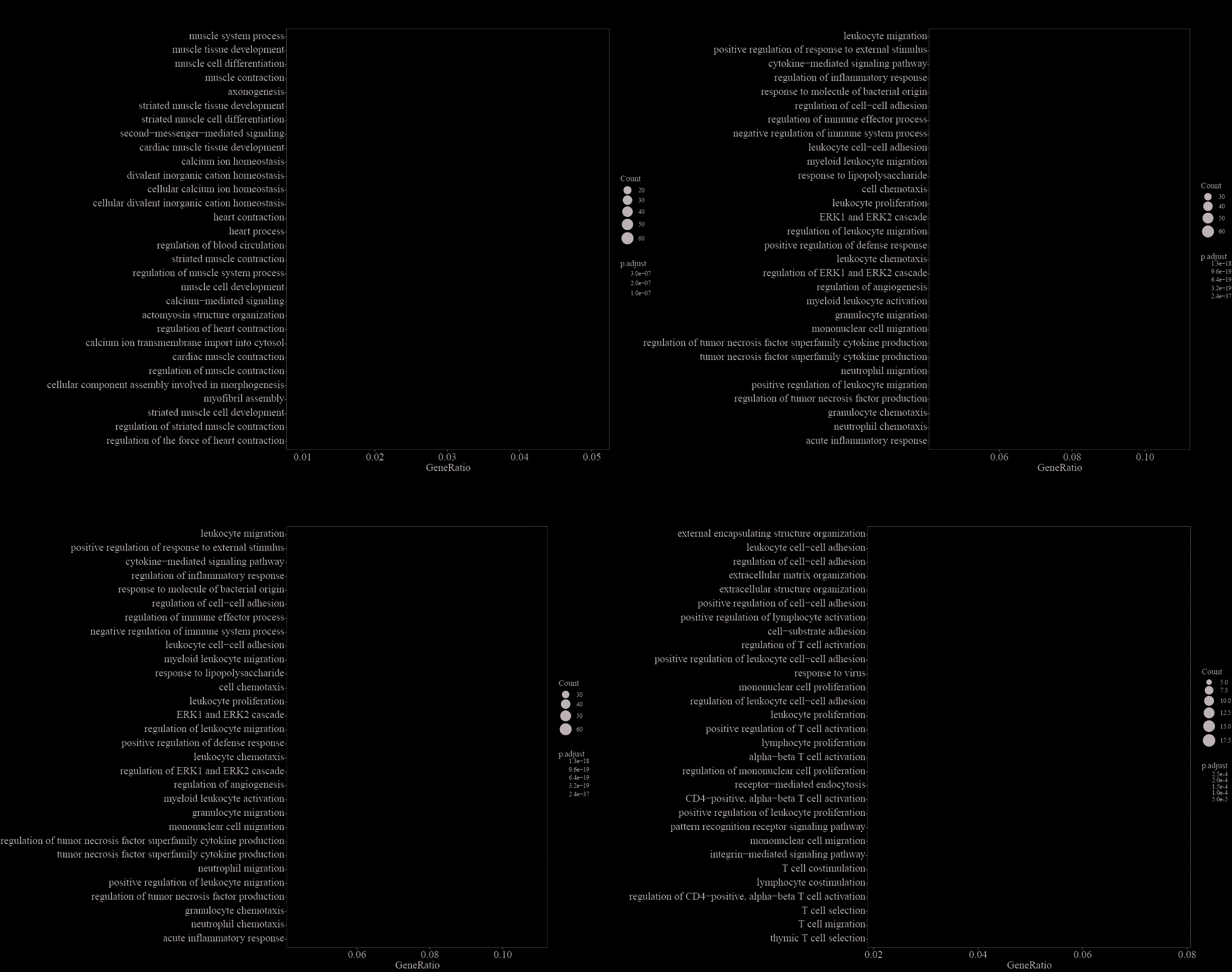

Supplement: Supplementary Figure 5 — Transcriptome sequencing results. (A) GO functional enrichment analysis of biological processes in the FRM versus FR groups. The left panel shows genes whose expression increased, and the right panel shows genes whose expression decreased. (B) GO functional enrichment analysis of biological process terms associated with FRM versus NFRM. The left figure shows the upregulation process, and the right figure shows the downregulation process. [file Image5.tif]

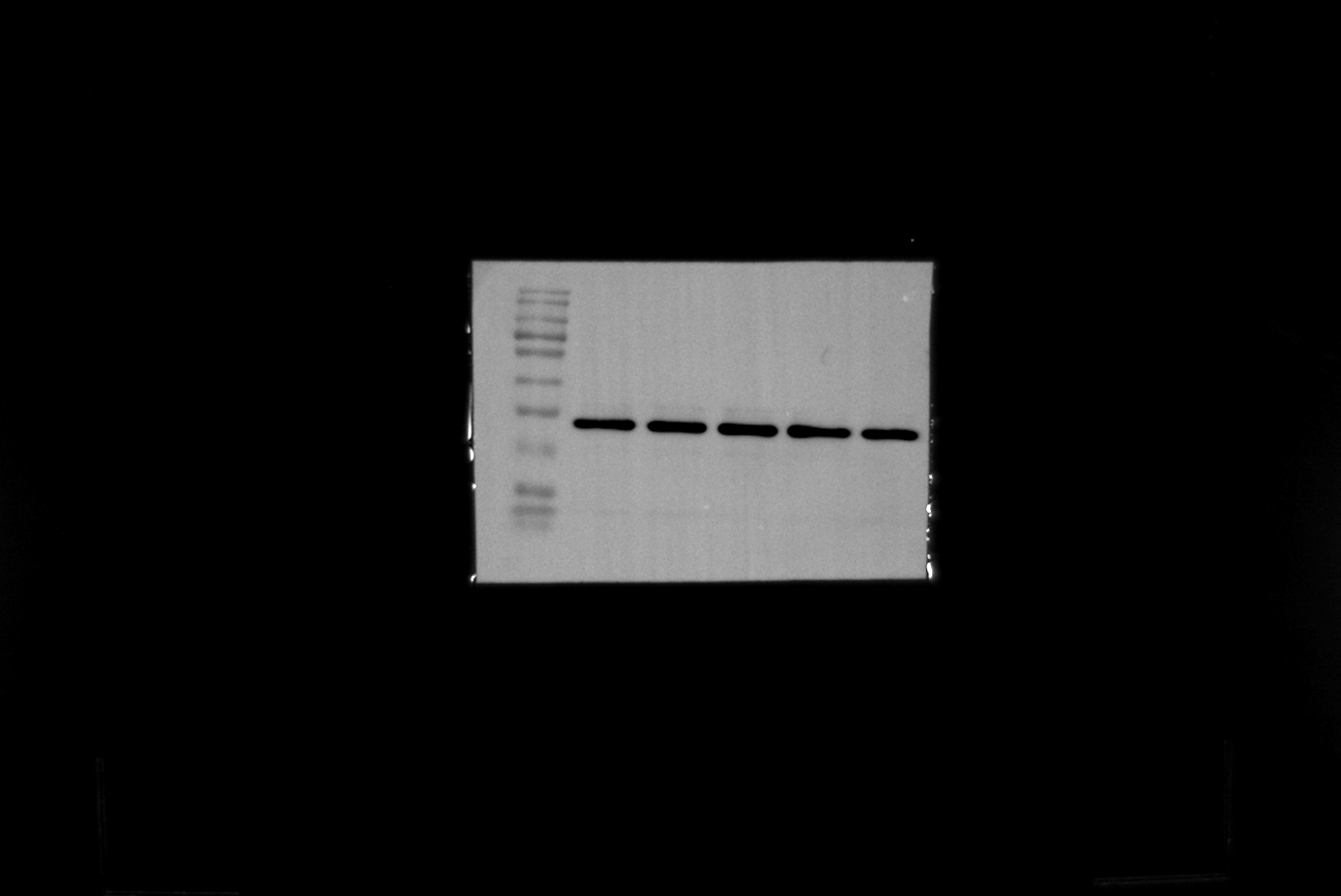

Supplement: Supplementary file 6 [file DataSheet1.zip › original data/wb-image/GAPDH.tif]

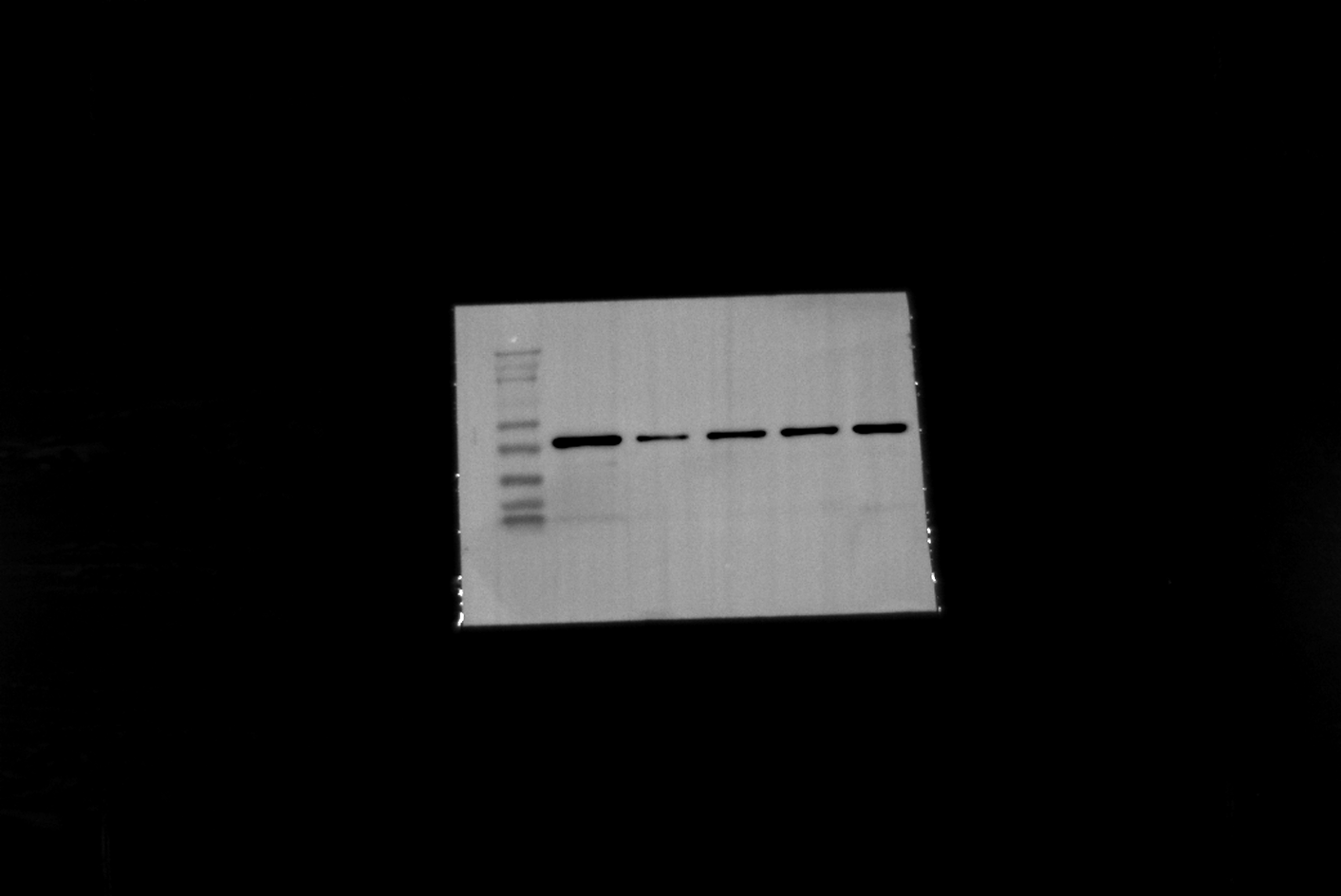

Supplement: Supplementary file 6 [file DataSheet1.zip › original data/wb-image/IKBA.tif]

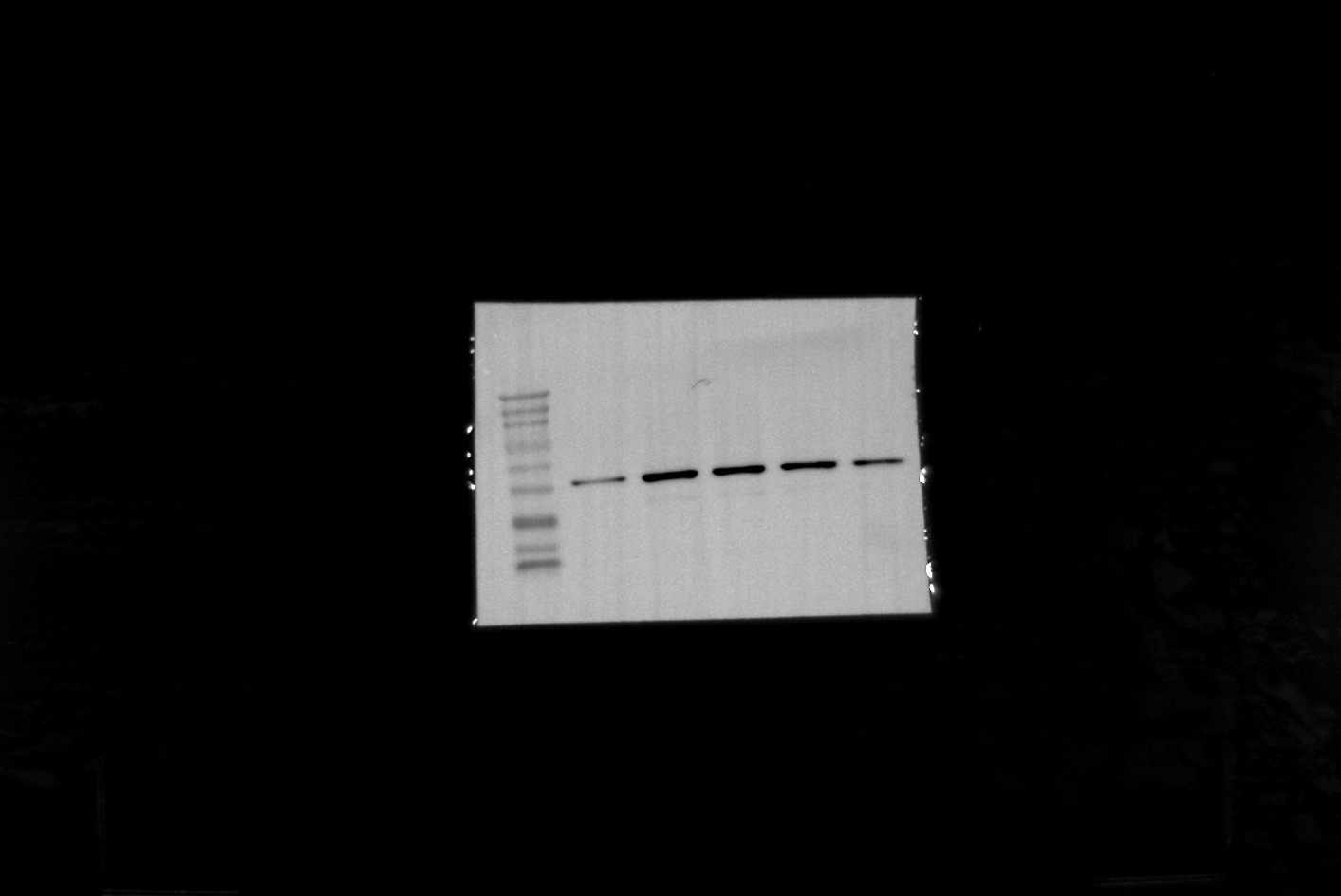

Supplement: Supplementary file 6 [file DataSheet1.zip › original data/wb-image/p-ikba.tif]

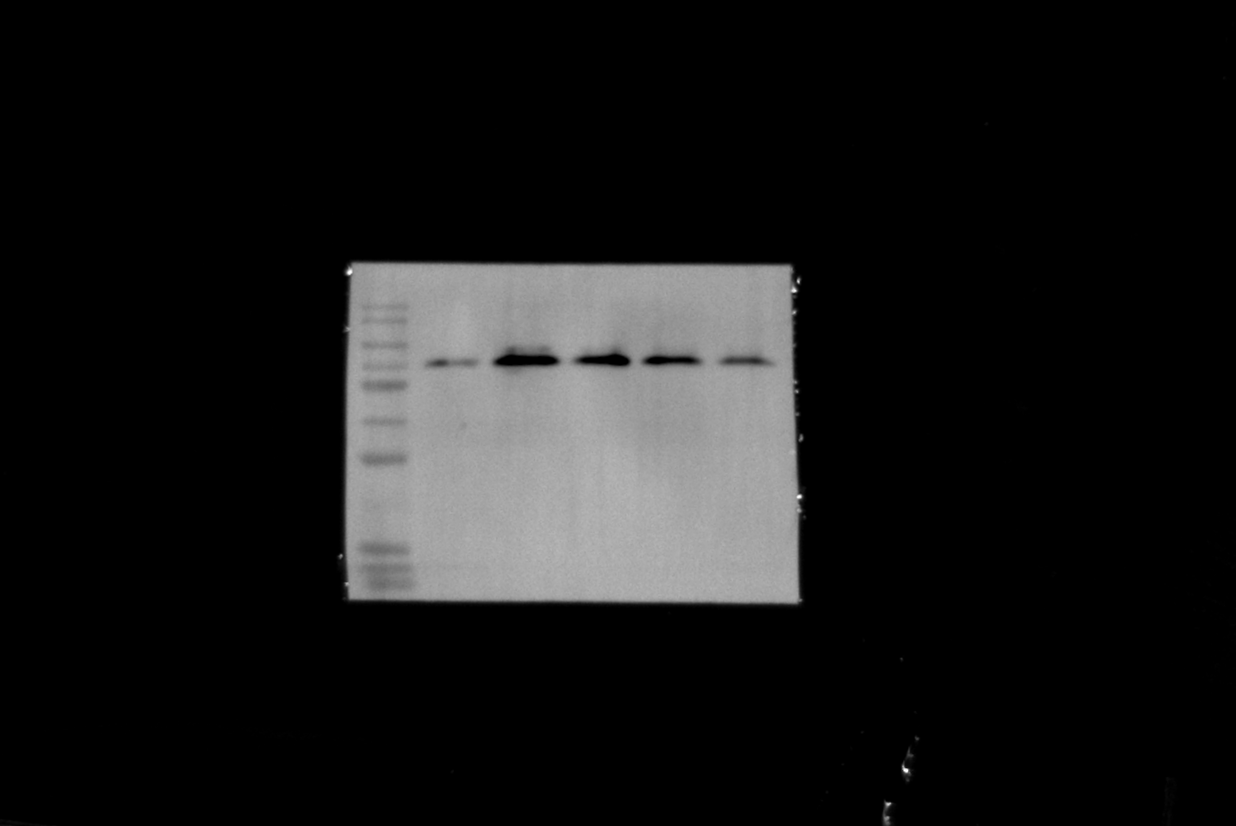

Supplement: Supplementary file 6 [file DataSheet1.zip › original data/wb-image/P-IKK.tif]

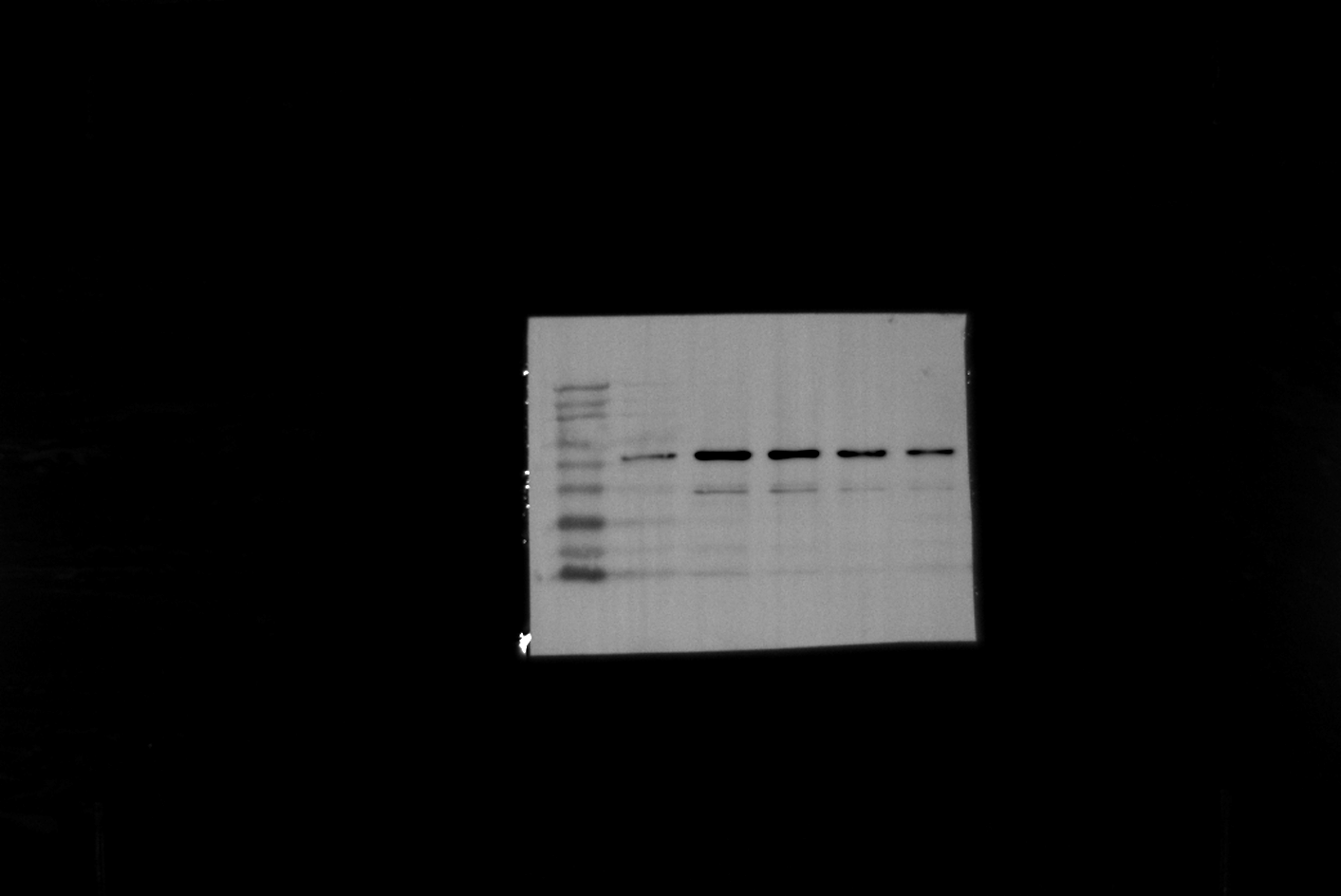

Supplement: Supplementary file 6 [file DataSheet1.zip › original data/wb-image/p-p65.tif]

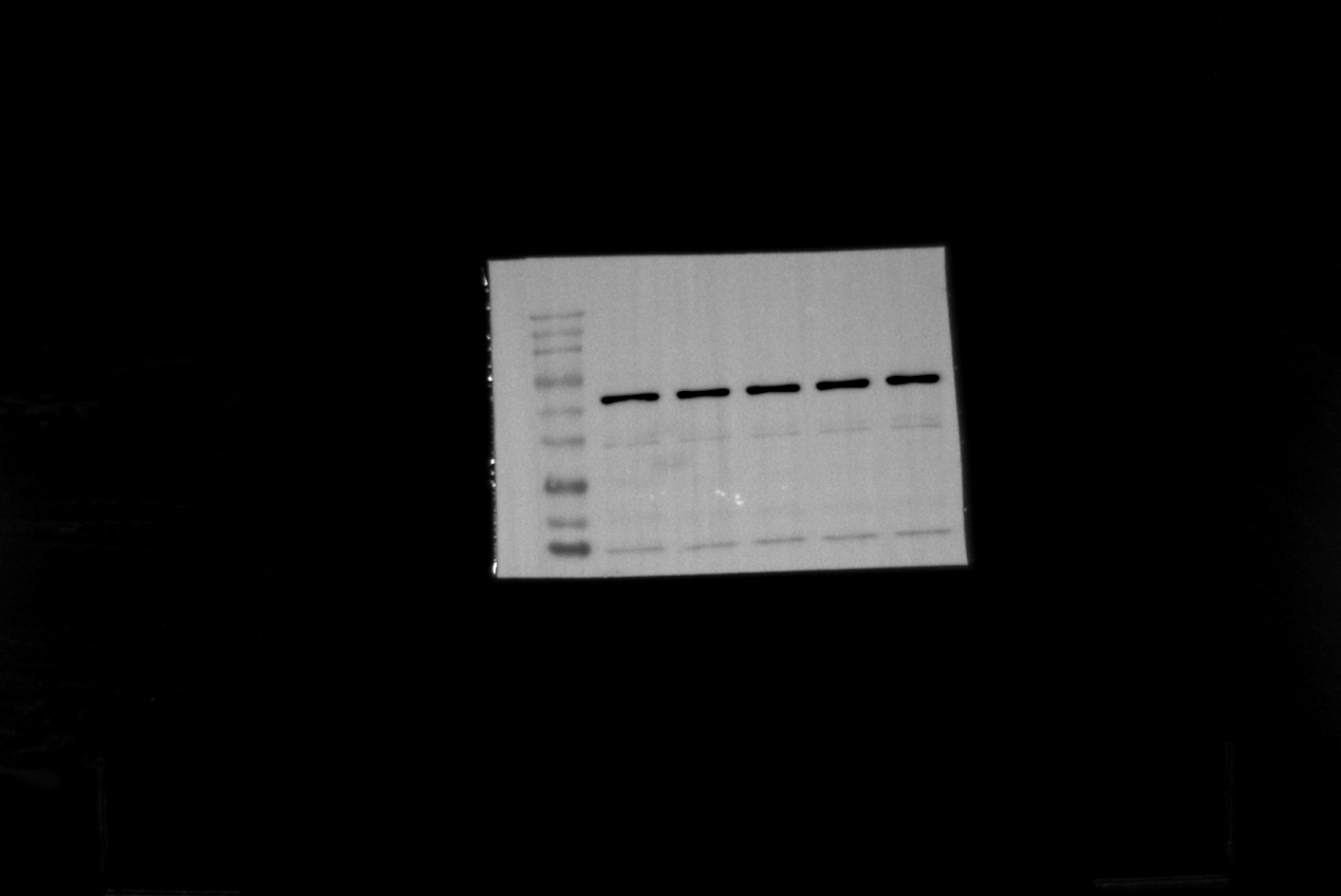

Supplement: Supplementary file 6 [file DataSheet1.zip › original data/wb-image/P65.tif]
