# Supplementary material for: Suppression of overactivated immunity in the early stage is the key to improve the prognosis in severe burns
Source: Front Immunol. 2024 Sep 6;15:1455899. doi: 10.3389/fimmu.2024.1455899 (PMC11412824; doi:10.3389/fimmu.2024.1455899)
Supplement: Supplementary file 7 [file Table1.pdf]

Table S1 Premiers for RT-qPCR of hub DEGs

GENE	Premier Sequence (5' → 3')
Homo sapine S100A8	F: ATGCCGTCTACAGGGATGAC<br>R: ACTGAGGACACTCGGTCTCTA
Homo sapine S100A9	F: GGTCATAGAACACATCATGGAGG<br>R: GGCCTGGCTTATGGTGGTG
Homo sapine S100A12	F: AGCATCTGGAGGGAATTGTCA<br>R: GCAATGGCTACCAGGGATATGAA
Homo sapine ITGAM	F: GCCTTGACCTTATGTCATGGG<br>R: CCTGTGCTGTAGTCGCACT
Homo sapine SPI1	F: GTGCCCTATGACACGGATCTA<br>R: AGTCCCAGTAATGGTCGCTAT
Homo sapine TLR8	F: GTGCCCTATGACACGGATCTA<br>R: AGTCCCAGTAATGGTCGCTAT
Homo sapine GAPDH	F: GGAGCGAGATCCCTCCAAAAT<br>R: GGCTGTTGTCATACTTCTCATGG
Mouse S100A8	F: AAATCACCATGCCCTCTACAAG<br>R: CCCACTTTTATCACCATCGCAA
Mouse GAPDH	F: AGGTCGGTGTGAACGGATTTG<br>R: GGGGTCGTTGATGGCAACA
