# Supplementary material for: Suppression of overactivated immunity in the early stage is the key to improve the prognosis in severe burns
Source: Front Immunol. 2024 Sep 6;15:1455899. doi: 10.3389/fimmu.2024.1455899 (PMC11412824; doi:10.3389/fimmu.2024.1455899)
Supplement: Supplementary file 8 [file Table2.pdf]

Table S2 Potential Drugs For Hub Genes

GENE	Drug	Interaction Type & Directionality	Sources	PMIDs	Query Score	Interaction Score
TLR8	MOTOLIMOD	agonist (activating)	TdgClinicalTrial TTD	None found	8.61	30.91
TLR8	CHEMBL512901	n/a	DTC	23899291	8.61	30.91
TLR8	RESIQUIMOD	agonist (activating)	DTC	24383475<br>16081189	6.46	23.19
TLR8	TELRATOLIMOD	n/a	TTD	None found	2.15	7.73
S100A9	TASQUINIMOD	n/a	TTD	24162378	8.61	61.83
S100A9	PAQUINIMOD	n/a	TTD	None found	4.3	30.91
S100A12	ATOGEPANT	n/a	TTD	None found	4.3	12.37
S100A12	RIMEGEPANT	n/a	TTD	None found	2.15	6.18
S100A12	UBROGEPANT	n/a	TTD	None found	2.15	6.18
S100A12	EPTINEZUMAB	n/a	TTD	None found	1.08	3.09
S100A12	METHOTREXATE	n/a	NCI	15077313	0.24	0.23
S100A8	METHOTREXATE	n/a	NCI	14722212	0.24	1.14
ITGAM	LIAROZOLE	n/a	NCI	9603657	2.87	4.12
ITGAM	ROVELIZUMAB	antagonist (inhibitory)	ChemblInteractions	None found	1.43	2.06
ITGAM	DIMETHYL<br>SULFOXIDE	n/a	NCI	15839205	1.43	2.06
ITGAM	FENTANYL	n/a	NCI	15098168	0.69	0.49
ITGAM	CLARITHROMYCIN	n/a	NCI	12167449	0.66	0.95
ITGAM	PHENYLEPHRINE	n/a	NCI	10973693	0.61	0.88
ITGAM	THEOPHYLLINE	n/a	NCI	9762784	0.36	0.52
ITGAM	MORPHINE	n/a	NCI	15098168	0.28	0.4
ITGAM	HYDROCORTISONE	n/a	NCI	18028766	0.23	0.33
ITGAM	ATORVASTATIN	n/a	NCI	18333374	0.14	0.21
